# Tailoring Robust 2D Nanochannels by Radical Polymerization for Efficient Molecular Sieving

**DOI:** 10.1002/advs.202409556

**Published:** 2024-12-31

**Authors:** Yue You, Yuxi Ma, Xianghui Zeng, Yichao Wang, Juan Du, Yijun Qian, Guoliang Yang, Yuyu Su, Weiwei Lei, Shuaifei Zhao, Yan Qing, Yiqiang Wu, Jingliang Li

**Affiliations:** ^1^ Institute for Frontier Materials Deakin University Geelong Victoria 3220 Australia; ^2^ Department of Applied Chemistry and Environmental Science School of Science RMIT University Melbourne Victoria 3000 Australia; ^3^ Faculty of Materials Wuhan University of Science & Technology Wuhan 430081 China; ^4^ Key Laboratory of Core Technology of High Specific Energy Battery and Key Materials for Petroleum and Chemical Industry College of Energy Soochow University Suzhou 215006 China; ^5^ Department of Chemical and Environmental Engineering, School of Engineering RMIT University Melbourne Victoria 3000 Australia; ^6^ College of Materials Science and Engineering Central South University of Forestry and Technology Changsha 410004 China

**Keywords:** 2D membranes, high water permeance, radical‐polymerization, robust nanochannels, water purification

## Abstract

Two‐dimensional (2D) nanochannels have demonstrated outstanding performance for sieving specific molecules or ions, owing to their uniform molecular channel sizes and interlayer physical/chemical properties. However, controllably tuning nanochannel spaces with specific sizes and simultaneously achieving high mechanical strength remain the main challenges. In this work, the inter‐sheet gallery d‐spacing of graphene oxide (GO) membrane is successfully tailored with high mechanical strength via a general radical‐induced polymerization strategy. The introduced amide groups from N‐Vinylformamide significantly reinforce the 2D nanochannels within the freestanding membranes, resulting in an ultrahigh tensile strength of up to 105 MPa. The d‐spacing of the membrane is controllably tuned within a range of 0.799–1.410 nm, resulting in a variable water permeance of up to 218 L m^−2^ h^−1^ bar^−1^ (1304% higher than that of the pristine GO membranes). In particular, the tailored membranes demonstrate excellent water permeance stability (140 L m^−2^ h^−1^ bar^−1^) in a 200‐h long‐term operation and high selectivity of solutes under harsh conditions, including a wide range of pH from 4.0 to 10.0, up to a loading pressure of 12 bar and an external temperature of 40 °C. This approach comprehensively achieves a balance between sieving performance and mechanical strength, satisfying the requirements for the next‐generation molecular sieving membranes.

## Graphical abstract

A general radical‐induced polymerization strategy to synthesize poly(N‐vinylformamide) short chains on GO nanosheets to tailor the gallery space of 2D nanochannels and build robust hydrogen‐bonded frameworks that help boost water permeance and reinforce the mechanical strength of the membranes.

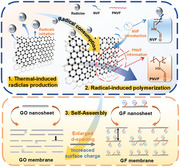

## 1 Introduction

The challenges in constructing selective pores/channels in water purification membranes, either conventional polymeric membranes or membranes made of 2D materials, that can achieve both a high water permeability and mechanical strength under realistic conditions remain unaddressed.^[^ ^1^, ^2^, ^3^ ^]^ Traditional polymeric membranes typically have robust selective pores but is hard to meet the high‐water permeability requirements for practical applications.^[^ ^4^, ^5^, ^6^, ^7^ ^]^ Compared to conventional polymeric membranes, membranes made of 2D materials are capable of achieving higher water permeances but their nanochannels have poor stability in aqueous solutions, especially under extreme pH conditions.^[^ ^8^, ^9^, ^10^, ^11^ ^]^ There is an urgent need for designing a new generation of 2D nanochannels that can separate small‐size molecules, achieve fast water permeation, and have superior mechanical properties under extremely harsh conditions.^[^ ^12^, ^13^, ^14^ ^]^

Graphene oxide (GO) membranes with 2D nanochannels^[^ ^15^, ^16^, ^17^, ^18^ ^]^ have attracted much interest due to their relatively high water permeability,^[^ ^19^, ^20^ ^]^ tunable ionic selectivity,^[^ ^21^, ^22^, ^23^ ^]^ high chemical resistance^[^ ^24^, ^25^, ^26^, ^27^, ^28^ ^]^ and potentially low manufacturing cost,^[^ ^23^, ^29^, ^30^ ^]^ compared with conventional polymeric membranes. All these merits are attributed to the unique physiochemical properties of GO membranes, including laminated structures^[^ ^31^, ^32^, ^33^ ^]^ and high surface charge originated from oxygen‐containing functional groups.^[^ ^34^ ^]^ However, GO membranes are not stable enough for long‐term operation, particularly under harsh conditions, and have slow water transport, which limits their practical applications.^[^ ^35^, ^36^ ^]^ The poor stability is primarily caused by membrane swelling induced by the ionization or deprotonation of the functional groups in the 2D nanochannels.^[^ ^37^, ^38^, ^39^ ^]^ In addition to poor stability, low water permeability of GO membranes is another serious concern. The water permeability of the reported GO membranes is still not high enough for practical applications, even though they have shown significantly faster water transport compared to traditional polymeric membranes.^[^ ^40^ ^]^

Numerous efforts have been made to build GO membranes with robust and fast water transport channels. Polymers like tetrakis (1‐methyl‐pyridinium‐4‐yl) porphyrin^[^ ^41^ ^]^ and polyethyleneimine^[^ ^42^ ^]^ were intercalated between GO nanosheets to simultaneously adjust the d‐spacing of GO membranes and link the local functional groups via electrostatic interactions and hydrogen bonds, resulting in a maximum water permeance of 20 L m^−2^ h^−1^ bar^−1^ (denoted as LMH bar^−1^) with improved stability and anti‐swelling property. However, the water permeance was still insufficient for practical applications. Intercalation of inorganic nanoparticles, such as ZnO nanoparticles between GO interlayers increased water permeance up to 225 LMH bar^−1^.^[^ ^43^ ^]^ However, the use of nanoparticles has safety concerns as they could be released into water.^[^ ^44^ ^]^ Forming robust hydrogen‐bonded (H‐bonded) frameworks within adjacent nanosheets could potentially enhance the mechanical properties and improve the selectivity of 2D membranes simultaneously.^[^ ^10^, ^45^ ^]^ However, achieving functional GO membranes with fast water permeation and high stability under harsh conditions is still challenging.

**Figure 1:**
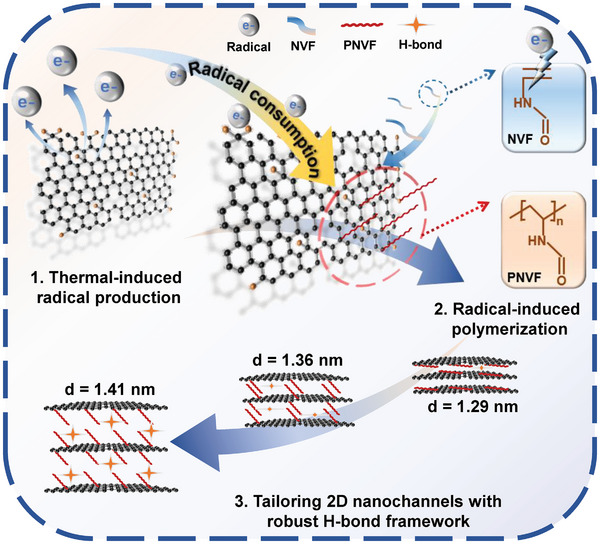
The schematic mechanism of tailoring 2D nanochannels via radical‐induced polymerization strategy.

In this work, we demonstrate a general radical‐induced polymerization strategy to synthesize poly(N‐vinyl formamide) (PNVF) short chains on GO nanosheets to tailor the gallery space of 2D nanochannels and build robust H‐bonded frameworks that help boost water permeance and reinforce the mechanical strength of the membranes. The radicals, namely unpaired valence electrons with well‐known high chemical activity, can be easily induced by energy infusions, including thermal, radiation, and redox reaction processes.^[^ ^46^, ^47^, ^48^, ^49^ ^]^ Attributed to the presence of abundant potential radical‐production sites, including various functional groups and defects on its aromatic plane, GO can be functionalized via radical‐induced polymerization. N‐vinylformamide (NVF), containing a reactive primary amide group and a vinyl group,^[^ ^50^ ^]^ was selected as a muti‐functional regulator to functionalize GO nanosheets and control the d‐spacing as well as the chemistry of the nanochannels in GO membranes. The d‐spacing of nanochannels could be controlled by adjusting the quantities of NVF monomers. The amide groups, on the side chains of PNVF, not only tuned the charge of the membrane nanochannels due to their negatively charged oxygen atoms,^[^ ^51^, ^52^, ^53^ ^]^ but also contributed to the formation of robust H‐bonded frameworks via the hydrogen donor (─NH─) and acceptor (─CO─) of amide groups.^[^ ^54^, ^55^, ^56^ ^]^ As shown in **Figure** **1**, the content of high‐activity radicals was optimized by adjusting both the heating temperature and duration. Then, the radicals propagated to the reaction sites on GO nanosheets through grabbing or sharing the electrons with neighboring molecules.^[^ ^57^ ^]^ Simultaneously, these radicals initiated the polymerization of monomers, resulting in the formation of PNVFs on GO nanosheets and robust H‐bonded frameworks within the channels. By adjusting the quantities of NVF, the channel size in GO/PNVF (GF) membranes were successfully tailored, resulting in a 13‐fold increase in water permeance compared with pristine GO membranes. This innovative method effectively strikes a balance between sieving performance and mechanical strength, meeting the requirements for the forthcoming generation of molecular sieving membranes.

## 2 Results and Discussion

### 2.1 Morphology and Chemical Composition of GF Nanosheets and Membranes

**Figure 2:**
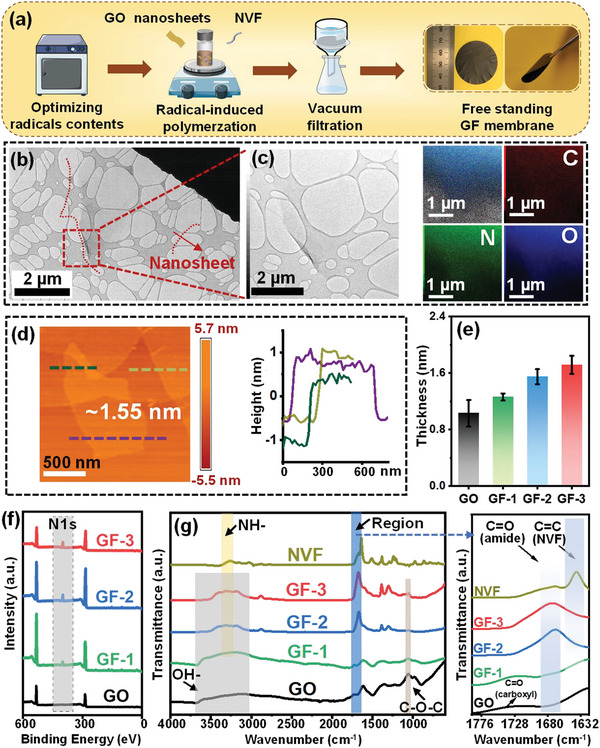
Characterization of the GO and GF nanosheets and membranes. a) Schematic illustration of fabricating a freestanding GF membrane. b) Bright field transmission electron microscopy image of GF‐2 nanosheets. c) Scanning transmission electron microscopy image of GF‐2 nanosheets. The insets are EDS mappings of GF nanosheets. d) Atom force microscope (AFM) images and height profile of GF‐2 nanosheets. e) Thickness distribution of GO and GF nanosheets. f) X‐ray photoelectron spectroscopy (XPS) survey spectra of GO and GF membranes. g) Fourier transform infrared (FTIR) spectra of NVF, GO, and GF membranes.

The process for preparing GF nanosheets and membranes is schematically illustrated in **Figure** **2**a. Uniform GF nanosheets and corresponding freestanding flexible GF membranes were produced via a general radical‐induced polymerization strategy by heating an aqueous dispersion of GO nanosheets and NVF monomers for 4 h at 95 °C. The use of this temperature and heating duration could produce the highest radical content on GO (Figures S1–S3 and Table S1, Supporting Information).

The morphology of pristine GO nanosheets and GF nanosheets were characterized by SEM and TEM (Figure 2b; Figures S4–S6, Supporting Information). As shown in Figure 2b, the transparent GF nanosheets had a 2D structure, similar to that of GO nanosheets. As shown in Figure 2d and Figure S7 (Supporting Information), the thickness of GF nanosheets increased from 1 nm (GO) to 1.26 nm (GF‐1), 1.55 nm (GF‐2), and 1.72 nm (GF‐3), respectively. The increase of thickness was due to the increasing amount of PNVF, which formed thin layers of uniform thicknesses on GO nanosheets.

The laminated structures of the GF and pristine GO membranes are shown in Figure S8 (Supporting Information). XPS was used to confirm the chemical composition of the membranes (Figure 2f; Figure S9, Supporting Information). The presence of nitrogen peaks in all GF membranes suggested the successful grafting of PNVF on GO nanosheets, consistent with the results from energy‐dispersive X‐ray spectroscopy (EDS) (Figure 2c; Figure S6, Supporting Information). Meanwhile, the relative atomic nitrogen percentage of GF membranes increased from 33.40% (GF‐1) to 34.92% (GF‐2) and 44.99% (GF‐3). The result indicates that an increasing amount of amide groups and hence NVFs were introduced on the surface of GO nanosheets with the increment of NVF loading in the radical‐induced polymerization process (Figure S9a, Supporting Information). The deconvoluted high‐resolution XPS spectra of N 1s revealed that the nitrogen configurations of GF membranes were at 399.6 and 401.3 eV, which can be assigned to N─(C═O)─ and C─N─H,^[^ ^58^, ^59^, ^60^ ^]^ respectively (Figure S9b–d, Supporting Information).

The crystalline structure and d‐spacing of GO and GF membranes were characterized by X‐ray diffraction (XRD) analysis, which showed that the main diffraction peak (001) of GO gradually shifted after the radical‐polymerization process (Figure S10, Supporting Information).^[^ ^23^ ^]^ The crystalline distortion along (001) direction was due to intercalations of PNVF chains within membrane nanochannels, indicating that the size of water transport pathways was successfully adjusted by tunning the loading of NVF monomers. Besides the d‐spacings, the local environment of functional groups within the nanochannels was also modified. As shown in Figure 2g, three main absorption peaks were found in the pristine GO membrane, which was assigned to C─O─C (≈1070 cm^−1^), −OH vibrations (from ≈3100 to ≈3700 cm^−1^), and the C═O (≈1728 cm^−1^) from the epoxy, hydroxyl, and carboxyl groups, respectively, of GO.^[^ ^61^, ^62^, ^63^ ^]^ For NVF monomers, the peaks of ─NH (≈3200 to ≈3300 cm^−1^)^[^ ^64^ ^]^ and amide I C═O (≈1668 cm^−1^)^[^ ^65^ ^]^ from amide groups and C═C (≈1646 cm^−1^)^[^ ^66^ ^]^ were evident. From GF‐1 to GF‐3 membranes, the gradual decrease of carboxyl C═O (≈1728 cm^−1^) and increase of the amide C═O (1670 cm^−1^), as well as the disappearance of the C═C peak (from NVF monomers) across all GF membranes proved that the polymerized NVF short chains were successfully grafted onto GO nanosheets (Figure S11, Supporting Information). Furthermore, the disappearance of C═C peak from NVF and C═O (≈1728 cm^−1^ carboxyl groups) peak from GO in the GF membrane with the higher amounts of NVF (GF‐2 and GF‐3) indicated that these two functional groups were fully consumed and formed C─O─C bonds to link GO and NVF chains together. Significantly, for the GF‐1 membrane, peaks of C═O from both amide groups (≈1670 cm^−1^) and carboxylic groups (≈1728 cm^−1^) were observed, indicating that the quantity of NVF monomers was not enough to fully consume the carboxyl groups of GO.

**Figure 3:**
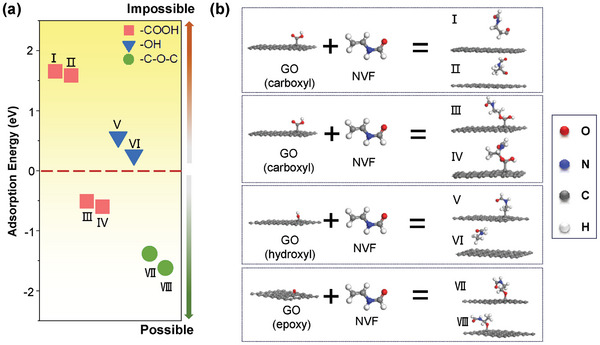
DFT calculations for the potential reaction sites between GO and NVF. a) Adsorption energy distribution, and b) corresponding models of reaction sites.

Density functional theory (DFT) calculation was performed to investigate the possible binding sites between the oxygen functional groups on GO and NVF monomers. The results revealed that the potential reaction sites toward NVF were as follows: epoxy (C─O─C in VII and VIII) > carboxyl (‐COOH in III and IV) > hydroxyl (‐OH in V and VI) > carboxyl (‐COOH in I and II) (**Figure** **3**). The negative adsorption energy values indicate an energetically feasible process.^[^ ^67^ ^]^ Thus, the DFT results indicated that carboxyl and epoxy were the main reaction sites, consistent with the FTIR characterization, which did not show significant changes in the absorption groups of epoxy. This is probably due to the consumption and regeneration of epoxy concurrently. Among all potential final bonding forms, the C─O─C bond formed favorably, linking PNVFs to GO nanosheets.

The transformation of oxygen‐containing functional groups was also supported by Raman spectroscopy. As shown in Raman spectra, the peak position of D and G bands remained the same at 1350 and 1600 cm^−1^ among all samples,^[^ ^68^ ^]^ except the width and shape (Figure S12, Supporting Information). The increments of I_D_/I_G_ from GO to GF revealed that the content of introduced oxygen groups from amide groups exceed the decomposed oxygen groups on GO nanosheets after the radical polymerization process.^[^ ^69^ ^]^ The quantities of functional groups on GF nanosheets were further evaluated by zeta potential measurements (Figure S13, Supporting Information). Compared with pristine GO nanosheets, the as‐prepared GF nanosheets had higher absolute zeta potential values. Among the as‐prepared GF nanosheets, GF‐2 balanced the trade‐off between the introduction of amide groups and the consumption of oxygen‐containing functional groups, all negatively charged, leading to a maximum absolute value of zeta potential.

### 2.2 Mechanical, Anti‐Swelling and Thermal‐Resistant Properties of GF Membranes

**Figure 4:**
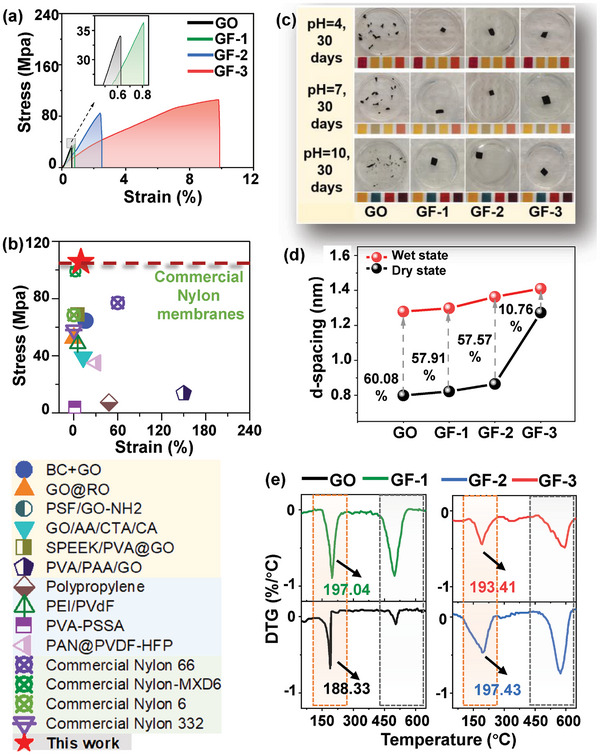
Mechanical properties of GO and GF membranes. a) Stress–strain curves of GO and GF membranes. b) A comparison of stress–strain value of some reported membranes and the membrane of this work. c) Photographs of GO and GF membranes immersed in DI water with pH 4, 7, and 10 for 30 days. d) Swelling ratios of GO and GF membranes. e) DTG spectra of GO and GF membranes.

As shown in **Figure** **4**a and Figures S14 and S15 (Supporting Information), the tensile strength of GF membranes was increased by 6.7% (GF‐1, 36 MPa), 148.4% (GF‐2, 84 MPa), and 210% (2 folds) (GF‐3, 105 MPa) over that of the GO membrane (34 MPa). Meantime, the strain of GF membranes was increased by 30.6% (GF‐1), 285% (GF‐2), and 1471% (15 folds) (GF‐3) compared to the GO membrane. The stress values of the GF membranes, the other reported GO‐based membranes and polymer‐based membranes are summarized in Figure 4b and Table S2 (Supporting Information). The GF‐3 membrane shows a much higher tensile stress value of up to 105 MPa, significantly higher than the membranes reported so far and even the commercial nylon membranes (max. 99 MPa).

Anti‐swelling ability is another crucial aspect for applications of 2D membranes. Compared with the pristine GO membranes, the GF membranes retained their integrity in aqueous solutions across a wide range of pH over 30 days, while the pristine GO membranes disintegrated into smaller pieces quickly, particularly in the alkaline solution (Figure 4c). To understand the changes of nanochannels, XRD was used to analyze the d‐spacings of wet membranes. The d‐spacing of the GF membranes exhibited a progressive increase with water immersion durations (Figure S16, Supporting Information). Notably, between 0.5 and 1 h, the d‐spacings of the GF membranes tended to stabilize. As shown in Figure S17 (Supporting Information), the d‐spacings of the membranes were respectively enlarged from 0.799 to 1.279 nm (pristine GO membrane), 0.822 to 1.289 nm (GF‐1 membrane), 0.865 to 1.363 nm (GF‐2 membrane) and 1.273 to 1.410 nm (GF‐3 membrane), while the swelling ratios decreased from 60.01% (GO) to 57.91% (GF‐1), 57.57% (GF‐2), and 10.76% (GF‐3) (Figure 4d). This is attributed to the introduction of PNVFs, which formed robust H‐bonded frameworks and linked adjacent GO nanosheets to resist swelling.

TGA and DTG characterizations were employed to compare the thermal stability of GO and GF membranes (Figure 4e; Figure S18, Supporting Information). For the pristine GO membranes, two distinct T_max_ values were observed, corresponding to different stages of thermal degradation. The first T_max_, between 100 and 360 °C, is attributed to the degradation of oxygen‐containing functional groups on the GO surface.^[^ ^70^ ^]^ The second T_max_, between 360 and 550 °C, corresponds to the oxidative pyrolysis of the carbon framework.^[^ ^70^ ^]^ The second T_max_ values of the GF membranes increased with the loading mass of NVF. This suggests that the attached PNVF chains enhanced the thermal stability of the membranes, as they underwent oxidative degradation at higher temperatures. The redshifts of the first T_max_ values among GF membranes indicate the increasing number of oxygen functional groups, consistent with the solution zeta potential results (Figure S13, Supporting Information). The thermal stability of the membranes is crucial for their long‐term performance, particularly in applications involving harsh conditions or high temperatures. The DTG data provide a basis for optimizing the membrane composition to achieve the desired balance between thermal stability and separation performance.

### 2.3 Membrane Separation Performance

**Figure 5:**
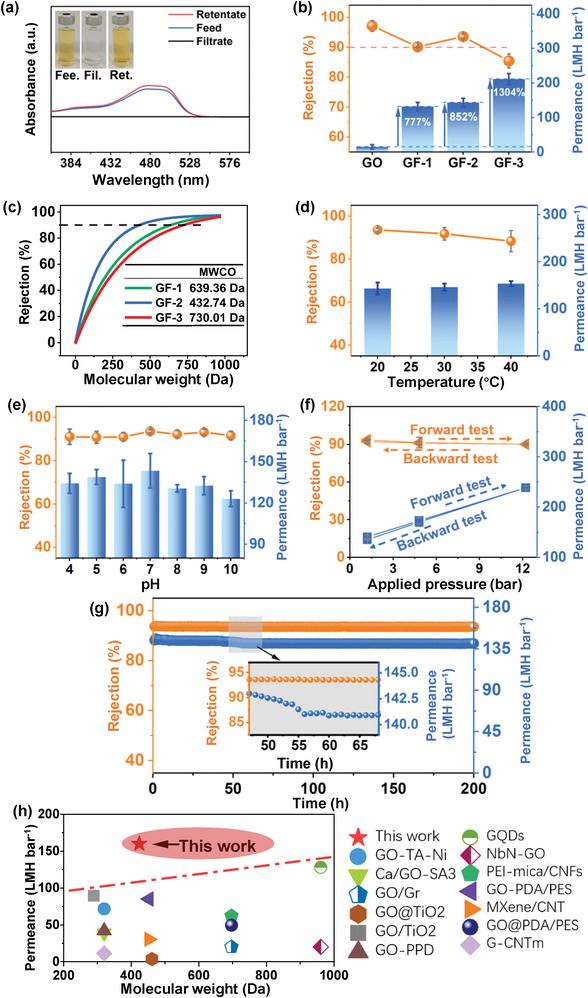
Separation performance of GO and GF membranes. a) UV–vis spectra of the feed, permeate, and retentate solutions of Orange G (OG) for GF‐2 membrane. The inset is a photograph of the solutions. The feed solution was 20 mL 10 mg L^−1^ OG, and the permeated side was filled with 20 mL of 0.05 m draw solution. b) Water permeance and OG rejection of GO and GF membranes. The feed solution was 20 mL 10 mg L^−1^ OG, and the permeated side was filled with 20 mL of 0.05 m draw solution. c) The molecular weight cutoff of GO and GF membranes is based on the rejections to dyes with different molecular weights. The feed solution was 20 mL 10 mg L^−1^ dye (*e.g*., MO, OG, EY, and RB), and the permeated side was filled with 20 mL of 0.05 m draw solution. d) Water permeance and OG rejection of the GF‐2 membrane under different temperatures. The feed solution was 20 mL 10 mg L^−1^ OG, and the permeated side was filled with 20 mL of 0.05 m draw solution. e) Water permeance and OG rejection of the GF‐2 membrane at different pH values. The feed solution was 20 mL 10 mg L^−1^ OG at different pH values, and the permeated side is filled with 20 mL of 0.05 m draw solution. f) Pressure loading tests for the separation performance of the GF‐2 membrane. The feed solution was 20 mL 10 mg L^−1^ OG, and the permeated side was filled with 20 mL of draw solution (e.g., 0.05, 0.2, and 0.5 m). g) Water permeance and OG rejection of the GF‐2 membrane over a 200‐h operating period. The feed solution was 20 mL 10 mg L^−1^ OG, and the permeated side was filled with 20 mL of 0.05 m draw solution. h) A comparison of water permeability of 2D water purification membranes (the selectivity, > 90%) that used different strategies to tune d‐spacings. All error bars represent standard deviations from the averages of three different measurements.

The water purification performance of the as‐prepared membranes for a series of anionic dyes, including Methyl Orange (MO), Orange G (OG), Eosin Yellow (EY), and Rose Bengal (RB), with different molecular weights was tested (Table S3, Supporting Information). As shown in **Figure** **5**a, the disappearance of color and the flattened UV spectrum of a typical permeate solution indicate that the OG molecules were efficiently removed by the GF‐2 membranes. The GO and GF membranes with the same mass loadings (2.36 mg cm^−2^) were further tested by filtering OG solutions through the membranes. The water permeance of the membranes significantly increased from 15.05 (GO membrane) to 132.09 (GF‐1), 143.35 (GF‐2) and 211.32 LMH bar^−1^ (GF‐3), corresponding to improvements by 777%, 852% and 1304%, respectively (Figure 5b). GF‐2 showed the highest rejection rate of 93.58%, which was slightly lower than that of the GO membrane (97.16%), among the three GF membranes. The results demonstrated that the GF‐2 membrane had a better trade‐off between water permeance and rejection rates. Moreover, all the as‐prepared GO and GF membranes were evaluated using MO, OG, EY, and RB dyes with different molecular weights (Figure S19, Supporting Information). The fitted molecular weight cut‐off (MWCO) of the GF‐1, GF‐2, and GF‐3 membranes (at the rejection ratio of 90%) were 639.36, 432.74, and 730.01 Da, respectively (Figure 5c).

The filtration performance of the GF‐2 membranes with increasing loading mass (from 1.18 to 4.72 mg cm^−2^) was further investigated to find the best membrane thickness for a good trade‐off between water permeance and rejection rate for the GF‐2 membranes (Figures S20 and S21, Supporting Information). With the increase in membrane thickness from 0.90 to 5.49 µm, the rejection rate for RB molecules was improved from 44.90% to 98.93%, while water permeance decreased from 236.27 to 109.63 LMH bar^−1^(Figure 5d; Figure S21, Supporting Information). A larger membrane thickness would increase the chance of retaining the solutes but slow down water transport.^[^ ^71^ ^]^ A membrane with a suitable thickness can achieve an optimal trade‐off between water permeance and dye rejection rate. In this work, the modulation of MWCO was achieved by membranes with the same amount of GF nanosheets, which had a variable surface zeta potential and thickness. The MWCO of a membrane is closely related to its structure and charges. The MWCO of GF‐2 (432.74 Da) is smaller than that of GF‐1 (639.36 Da) and GF‐3 (730.01 Da) due to differences in their d‐spacing and charges. The separation mechanisms are based on Donnan effects and steric hindrance, which are to be discussed in detail later. Specifically, both GF‐1 and GF‐3 have lower charges, while GF‐1 has a smaller d‐spacing and GF‐3 has a larger d‐spacing. GF‐2 has intermediate d‐spacing and the largest charges among them. The role of Donnan effects is more dominant than steric hindrance, thus GF‐2 has the smallest MWCO than those of GF‐1 and GF‐3. Therefore, the membrane with a loading mass of 2.36 mg cm^−2^ achieved a good balance between water permeance (166.53 LMH bar^−1^) and RB rejection rate (97.24%).

To simulate the harsh application environments, the influence of temperature (20–40 °C), pH (4.0‐10.0), and pressure (1.2‐12 bar) on the filtration performance of the GF‐2 membrane was investigated. With an increase in temperature, the water permeance slightly increased due to faster molecular diffusion, without significantly decrease in the rejection rate (Figure 5d).^[^ ^72^ ^]^ In the wide range of pH, especially in alkaline conditions (pH > 7.0), the GF‐2 membrane maintained high rejection rates, effectively overcoming the inherent instability of GO membranes in alkaline environments (Figure 5e). Additionally, poor pressure resistance is a significant concern for GO membranes and other 2D membranes in practical conditions. A positive linear relationship between water permeance and applied pressure either in the forward (pressure increase) or backward (pressure decrease) testing was obtained (Figure 5f). The rejection rate remained almost constant during the two‐way testing. The results suggested the high‐pressure resistance (up to 12 bar) of the GF‐2 membrane nanochannels, which shall fully meet the pressure requirements (usually 5–10 bar) for practical applications.^[^ ^73^ ^]^

The long‐term stability of the GF‐2 membrane was conducted over a duration of 200 h with feeds of OG solutions. Throughout the entire testing period, the GF‐2 membrane consistently demonstrated a high rejection rate (> 95%) for OG molecules (Figure 5g). The membranes maintained a high water permeance, with a very slight decrease from 143 to 141 LMH bar^−1^, between 50 and 55 h, and high selectivity for OG molecules. The results suggest that the GF membranes have super‐stable nanochannels and good antifouling ability. The water purification performance of other currently reported composite membranes with different channel tuning strategies is shown in Figure 5h and Table S4 (Supporting Information). The radical‐induced polymerization strategy enabled the breakage of the current trade‐off between water permeability and selectivity of GO membranes. Furthermore, the GF membranes with variable MWCO showed a potential for precisely sieving molecules with different molecular weights. Therefore, the combination of ultrahigh water permeance with adjustable MWCO and robust stability in diverse challenging conditions such as temperature, pH, and applied pressure, comprehensively demonstrated the potential suitability of GF membranes for practical applications.

### 2.4 Separation Mechanism

**Figure 6:**
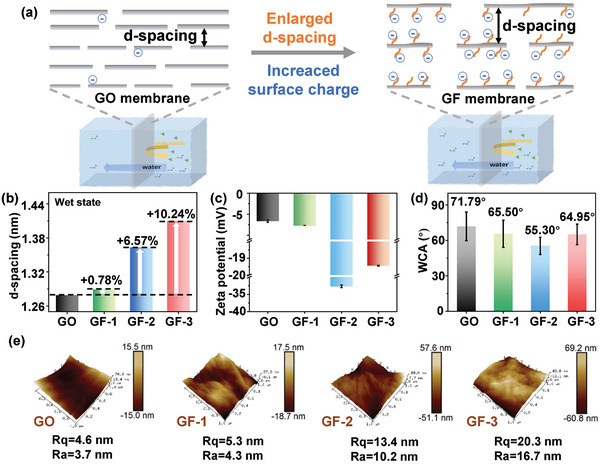
Separation mechanism of GF membranes. a) Scheme of the separation process by GO and GF membranes. b) The d‐spacing of GO and GF membranes in the wet state. c) Zeta potential of GO and GF membranes (pH 7.0). d) Water contact angles (WCA) of GO and GF membranes, and e) AFM images of surface roughness of GO and GF membranes.

The separation mechanism for pristine GO and GF membranes was schematically described in **Figure** **6**a. Both the Donnan effect and steric hindrance play key roles in maintaining membrane selectivity. The Donnan effect arises from the distribution of charges across the membrane, driven by charge repulsion between the membrane surface and dye molecules in solution. For charged dyes, the electrostatic interactions (due to the membrane's surface charge) result in selective dye rejection, particularly for similarly charged species. This charge‐based exclusion mechanism significantly impacts the transport of charged solutes, as demonstrated by the improved rejection of dyes based on charge repulsion. Moreover, steric effects are related to the physical size of the membrane channels. As the channel size increases, one might expect selectivity to decrease; however, a balance between permeability and selectivity can be maintained by optimizing surface charge. The interplay between nanochannel size and charge exclusion ensures that the selectivity of dyes does not decrease significantly, even as permeability increases. By introducing PNVF chains into nanochannels, both the size and surface charge of nanochannels in GF membranes were enlarged. In wet states, the d‐spacing of the GF membranes was about 0.78% (GF‐1), 6.57% (GF‐2), and 10.24% (GF‐3) larger than the pristine GO membranes. This enlarged lateral pathways provide more spaces for the movement of free water along a certain direction when an osmotic pressure is applied. As a result, it is expected that an increasing number of solutes would also be able to pass through the enlarged nanochannels due to the steric effect, consistent with the higher water permeance of the GF membranes. However, compared with the pristine GO membranes, the selectivity of the GF membranes for OG molecules did not experience a significant reduction due to the Donnan effect. The Donnan effect is determined by the chemistry of the nanochannels, and the surface charge of a membrane.^[^ ^74^ ^]^

Compared to GF‐2, although GF‐3 membrane had better thermal stability and mechanical properties, it had a smaller negative surface charge due to the consumption of more oxygen‐containing groups on its GO sheets for NVF grafting, resulting in a weaker Donnan effect. In contrast, GF‐2 membrane had the maximum oxygen group content via a balance between the consumption of oxygen groups on GO and the increment of oxygen groups from NVF. During radical‐polymerization on the GO surface, two key processes occurred: 1) the consumption of oxygen‐containing functional groups (which are responsible for negative charges on the GO surface, and 2) the introduction of amide groups from NVF, which also contribute negative charges. Although GF‐3 had more amide groups than GF‐2, the higher consumption of oxygen‐containing groups in GF‐3 reduced its overall negative charge compared to GF‐2. Consequently, GF‐3 exhibited fewer negative charges and thus had a higher zeta potential than GF‐2.

As shown in the membrane surface zeta potential results, the GF membranes exhibited more negative zeta‐potentials, which were −7.67 mV (GF‐1), −32.68 mV (GF‐2), and −19.44 mV (GF‐3), compared to the pristine GO membrane (‐6.64 mV) (Figure 6c). A higher negative charge of a membrane will help reject the negatively charged dyes due to electrostatic repulsion. The highest absolute zeta potential of the GF‐2 membrane contributed to the highest dye rejection among the GF membranes. However, as shown in Figure 6b, the pristine GO membrane exhibited a slightly higher dye rejection, attributed to its smaller d‐spacing compared to the size of OG molecules (0.54 × 1.01 × 1.56 nm^3^).^[^ ^75^ ^]^ This smaller d‐spacing is also the primary barrier, limiting the water permeance in the pristine GO membranes.

The enhanced water permeability is also affected by the introduced hydrophilic functional groups (amide groups) within the 2D nanochannels after NVF chain grafting. Although during the polymerization process, GO could be partially reduced to remove some oxygen‐containing group (negatively charged) due to the use of a moderately high temperature (95 °C),^[^ ^76^ ^]^ the membranes with PNVF were more hydrophilic (Figure 6d; Figure S22, Supporting Information), demonstrated by their smaller water contact angles compared to the pristine GO membrane. Increased surface roughness is another factor to enhances the membrane water permeability as it enlarges the effect filtration area during water permeation.^[^ ^77^ ^]^ As shown in Figure 6e, Table S5, and Figure S23 (Supporting Information), the GF‐3 membrane had the highest surface roughness while balancing the channel size and surface charge and exhibited the highest water permeability.

## 3 Conclusion

In conclusion, a simple thermal‐induced radical polymerization strategy was applied to construct robust nanochannels with variable sizes. The radicals on GO facilitated the breakage of C═C and C═O bonds, promoting the formation of C─O─C bonds and the linkage of GO nanosheets and NVF chains. Consequently, the enlarged charge and size of the inner gallery of the membranes led to the high water permeance of up to 218 LMH bar^−1^ with variable molecular MWCO from 432.74 to 730.01 Da, attributed to the steric and Donan effects of the GF membranes. Meanwhile, the introduction of NVF chains simultaneously reinforced the H‐bonded frameworks within nanochannels, enhancing the mechanical strength and stability of the membranes in aqueous solutions, even under harsh conditions for a long duration. This strategy provides not only a new method to adjust the nanochannels but also a potential avenue for breaking the current trade‐off between permeability and selectivity for water purification membranes.

## Conflict of Interest

The authors declare no conflict of interest.

## Supporting information



Supporting Information

## Data Availability

The data that support the findings of this study are available from the corresponding author upon reasonable request.
