# Supplementary material for: Tailoring Robust 2D Nanochannels by Radical Polymerization for Efficient Molecular Sieving
Source: Adv Sci (Weinh). 2024 Dec 31;12(8):2409556. doi: 10.1002/advs.202409556 (PMC11848538; doi:10.1002/advs.202409556)
Supplement: Supplementary file 1 — Supporting Information [file ADVS-12-2409556-s001.docx]

**Tailoring Robust 2D Nanochannels by Radical Polymerization for Efficient Molecular Sieving**

Yue You^[1]^, Yuxi Ma^[2]^, Xianghui Zeng^[3]^, Yichao Wang^[2]^, Juan Du^[1]^, Yijun Qian^[4]^, Guoliang Yang^[2]^, Yuyu Su^[6]^, Weiwei Lei^[2]*^, Shuaifei Zhao^[1]*^, Yan Qing^[5]^, Yiqiang Wu^[5]^, Jingliang Li^[1]*^

^[1]^ Institute for Frontier Materials, Deakin University, Geelong, Victoria 3220, Australia

^[2]^ Department of Applied Chemistry and Environmental Science, School of Science, RMIT University, Melbourne, Victoria 3000, Australia

^[3]^ Faculty of Materials, Wuhan University of Science & Technology, Wuhan 430081, China

^[4]^ Key Laboratory of Core Technology of High Specific Energy Battery and Key Materials for Petroleum and Chemical Industry, College of Energy, Soochow University, Suzhou 215006, China

^[5]^ College of Materials Science and Engineering, Central South University of Forestry and Technology, Changsha 410004, China

^[6]^ Department of Chemical and Environmental Engineering, School of Engineering, RMIT University, Melbourne, Victoria 3000, Australia

E-mail: weiwei.lei@rmit.edu.au; [s.zhao@deakin.edu.au](mailto:s.zhao@deakin.edu.au); [jingliang.li@deakin.edu.au](mailto:jingliang.li@deakin.edu.au)

Contents

[Supplementary Materials and Methods 4](#_Toc169878935)

[S1. Materials 4](#_Toc169878936)

[S2. Preparation of graphene oxide 4](#_Toc169878937)

[S3. Grafting PNVF on GO nanosheets by radical polymerization 4](#_Toc169878938)

[S4. Preparation of free-standing GF membranes 4](#_Toc169878939)

[S5. Characterization 5](#_Toc169878940)

[S6. Evaluation of water purification performance 6](#_Toc169878941)

[Supplementary Figures 7](#_Toc169878942)

[Supplementary Tables 30](#_Toc169878943)

[References 36](#_Toc169878944)

# **Supplementary Materials and Methods**

## **S1. Materials**

N-Vinylformamide (NVF, 98%), graphite powder (<20 μm), sulphuric acid (H_2_SO_4_, 95–97%), Methyl Orange (MO), Orange G (OG), Eosin Yellow (EY), Rose Bengal (RB), hydrochloric acid (HCl, 37%), hydrogen peroxide (H_2_O_2_, 30%), potassium permanganate (KMnO_4_, >99%), dialysis tubing (CelluSep T1/Nominal MWCO: 3,500), and nylon substrate membrane (200 nm pore size) and were purchased from Sigma-Aldrich and used as received.

## **S2. Preparation of graphene oxide**

Graphite oxide (GO) was fabricated using the modified Hummers method^[1]^. Briefly, 6 g of graphite powder was mixed with 240 mL of H_2_SO_4_ (98%). Subsequently, 24 g of KMnO_4_ was gradually added in an ice bath. Following this, the mixture stirred for 2 h at 35 °C. Afterwards, ultrapure water (700 mL) and H_2_O_2_ (60 mL) were added dropwise into the mixture. After an additional 2-h stirring, the mixture was filtered and washed with HCl aqueous solution (10%). Then, the GO suspension was diluted and dialyzed (5030-46 - dialysis tubing CelluSep T1/Nominal MWCO: 3,500) for 7 days. At last, the GO suspension in ultrapure water was exfoliated in a sonication bath for 60 min and then centrifuged at 3500 rpm for 60 min.

## **S3. Grafting PNVF on GO nanosheets by radical polymerization**

GO nanosheet suspensions (0.2 mg/ml, 10 ml) were mixed with NVF monomers at different mass ratios (GO : NVF) of 2:1, 1:1 and 1:2, respectively. Then, the suspensions were heated at 95 °C in an oil bath for 4 h to graft PNVF on GO nanosheet. The grafted nanosheets are denoted by GF-1, GF-2 and GF-3.

## **S4. Preparation of free-standing GF membranes**

The GF suspensions were vacuum (1 bar) filtrated on a battery separator substrate membrane (Celgard 3501-0650M-R). After vacuum filtration, the membrane was peeled off from the substrate and dried for 0.5 h in a fume hood (50 ± 5% relative humidity and 20 ± 3 °C). The membranes are denoted by GO membrane, GF-1 membrane, GF-2 membrane, and GF-3 membrane, respectively, corresponding to the different GO/NVF mass ratios. The volume of the GF suspensions determined the loading mass/thickness of the membrane.

## **S5. Characterization**

The morphologies and structures of GF nanosheets and membranes were observed using transmission electron microscopy (TEM, JEOL 2100), scanning electron microscopy (SEM, Zeiss Supra 55VP), and atomic force microscopy (AFM, Bruker multimode 8).

X-ray powder diffraction (XRD) measurements in the 2θ range from 5° to 20° (with a step size of 0.006° and recording rate of 150 ms) for GO and GF membranes in the dry state and hydrated state were performed using a Xpert Powder with Cu Kα radiation (λ = 1.5406 Å).

Raman spectra of GO and GF was obtained using a 325 nm green line of argon laser source (0.78 eV) and a power of 20 mW in the range of 300–3000 cm^−1^.

The X-ray photoelectron spectroscopy (XPS) was carried out using ESCALAB 250Xi.

Zeta potentials of GO and GF nanosheets or membranes were obtained by using a Malvern Zetasizer Nano and SurPass 3 Zeta Potential (Anton Paar) respectively. The elemental composition was determined with a CHN elemental analyzer (Elementar Vario EL; Thermo Fisher).

The surface functional groups of GO and GF membranes were characterized by Fourier transform infrared spectroscopy (Bruker Vertex 70) in the wavenumber 4000–500 cm^−1^ using a Thermo Fisher Nicolet iS 5.

A Micro-ESRTM (Active Spectrum, Inc., Foster City, CA) was used to measure the radical contents of GO samples. The parameters were as follows: a microwave frequency of 9.7 GHz, a power of 10 mW, and a mod coil amplitude of 10%. The results were averages of triplicate measurements after ten scans of each sample.

The water contact angles of GO and GF membranes were characterized using CAM101 (KSV Instruments Ltd.).

Thermogravimetric analysis (TGA 8000) of GO and GF membranes was characterized from 30 °C to 800 °C (rate of 10 °C min^−1^) at an N_2_ flow (20 mL min^−1^).

The tensile strength of GO and GF membranes was measured at 25 °C via a universal test machine (Instron 30 KN tensile tester) with a 5 N load cell. For strain cycles, all samples were stretched at a loading rate of 2 mm⋅min^−1^. The results were averages of triplicate measurements.

## **S6. Evaluation of water purification performance**

The water permeance (Jw) and the dye rejection (R) were obtained in forward osmosis mode, using a H-Cell device as shown in Figure S24 in the Supporting Document, consistent with our previous work^[2]^. The feed side was dye solution (20 mL,10 mg L^-1^) and the draw side was introduced the large-size molecule (20 mL, 0.05 M). The dye and water molecules can permeate into the draw cell. Therefore, this method is appropriate for the evaluation of the water purification performance of GO and GF membranes. To prevent concentration polarization, magnetic stirring at a speed of 1000 rpm was applied in both compartments simultaneously. In the long-term filtration experiments, the concentration of the feed solution was measured again after the fresh dye solution was injected. Each set of experiments was performed three times and the data obtained were averaged. The mass (mp) of water in permeated side was weighed on an analytical balance (HR-250AZ Compat, A&D Company Limited). The water permeance (Jw) was calculated using the following equations (1) and (2).

J_w_=$v_{P}/(t\times A\times p)$ (1)

V_p_=$m_{P}/\rho$ (2)

where Vp is the permeate volume (L) in a certain time t (h), A is the effective membrane area (m^2^), and P is pressure (bar), m_p_ is the mass (g) of permeate water, and ρ is water density (1×10^3^ g L^-1^). According to the Van’t Hoff equation, the 0.05 M draw solution can produce an osmotic pressure of ∼1.2 bar at the room temperature^[3]^.

The rejection (R) is calculated as Eq. (3).

R=(1-C_p_/C_f_) × 100% (3)

where C_p_ and C_f_, are the solute concentrations (mg L^-1^) of the permeate and feed solution, respectively.

# **Supplementary Figures**


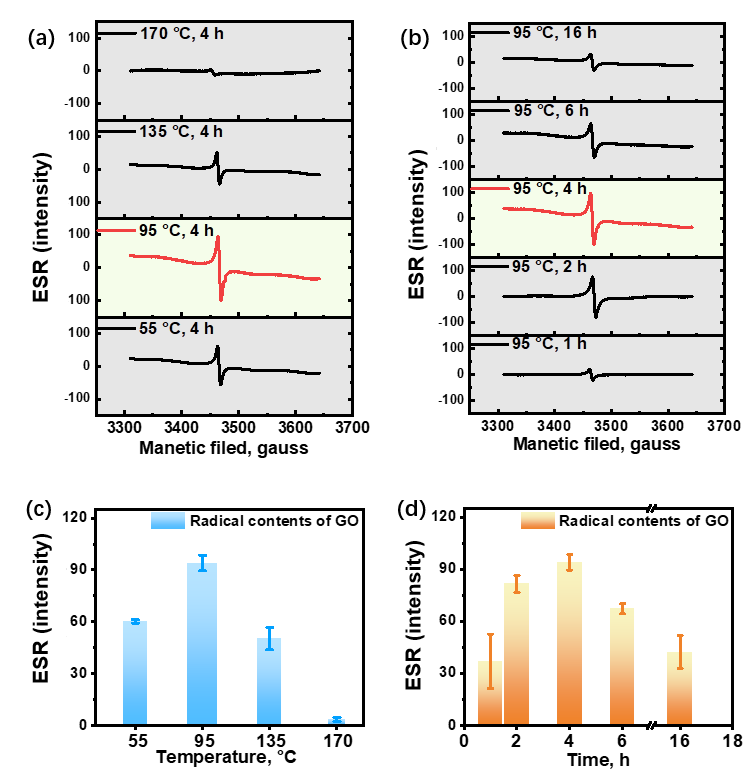


**Figure S1.** ESR spectra of GO nanosheet powder to characterize radicals produced by different **(a, c)** heating temperatures, and **(b, d)** heating time periods.


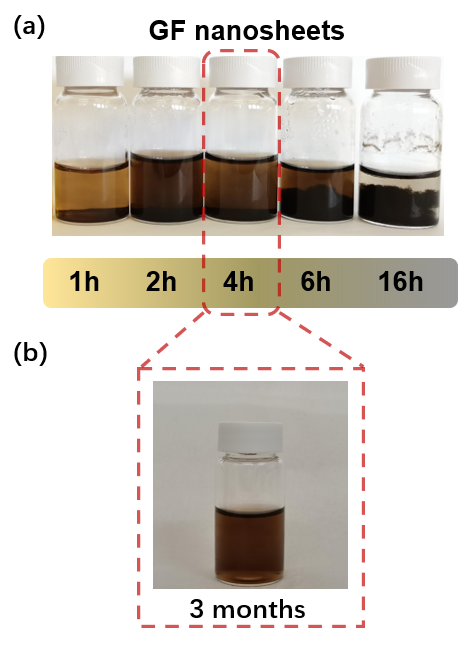


**Figure S2.** The image of **(a)** GF nanosheet with different reaction time, and the image of **(b)** the suspension of GF nanosheets (4h) stored in room temperature over 3 months.


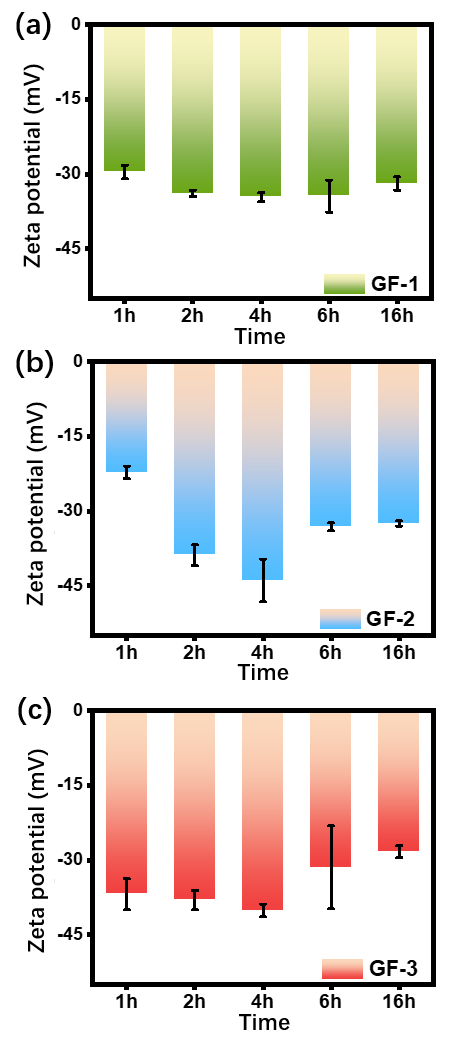


**Figure S3.** Zeta potentials of **(a)** GF-1, **(b)** GF-2, and **(c)** GF-3 nanosheets in DI water after different reaction period.


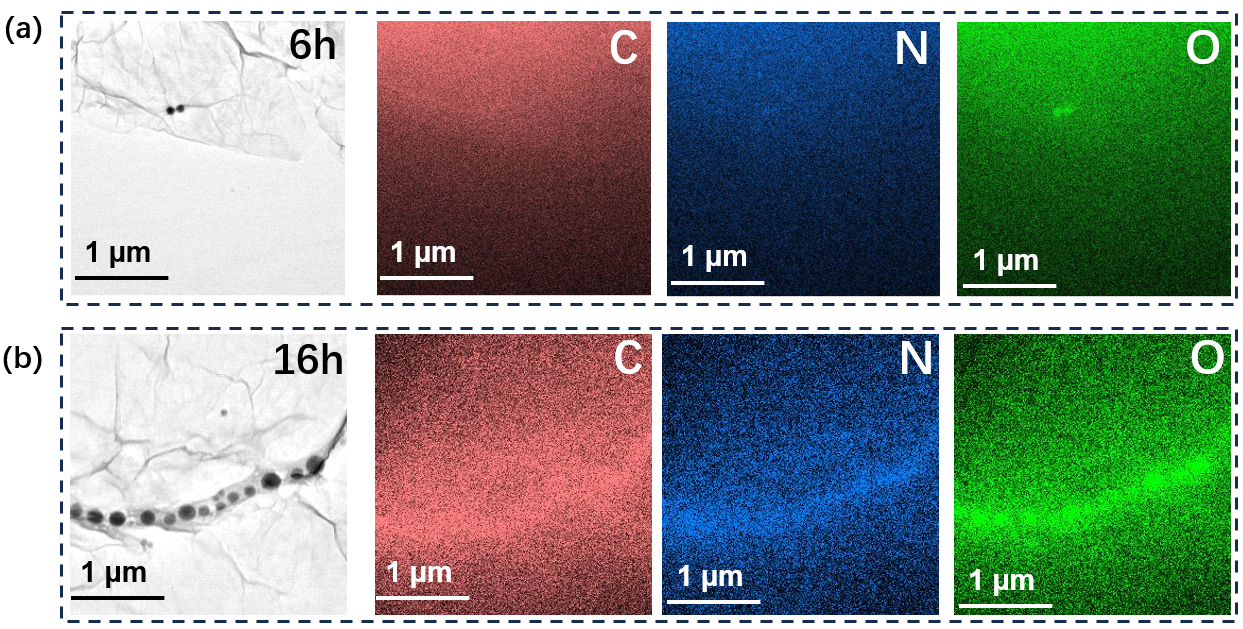


**Figure S4.** Scanning transmission electron microscopy (STEM) images of **(a)** GF-6h nanosheets and **(b)** GF-16h nanosheets.

To achieve efficient polymerization on GO nanosheets, the radical content of the nanosheets was optimized. Thermal treatment at 95 °C for 4 h led to the maximum radical content on GO nanosheets. Hence, this temperature and duration were selected for the polymerization. As shown in the Electron Spin Resonance (ESR) spectra, an optimal thermal temperature of 95 °C was selected to efficiently generate the maximum content of carbon-cantered radicals^[4]^ on GO (Figure S1 and Table S1). Then, the uniform GF dispersion was produced via thermal-induced radical-assisted polymerization by heating for 4 h at 95 °C. The color of the GF dispersion gradually became darker with the increase of the thermal treatment period (Figure S2). However, when the reaction duration exceeded 4 h, phase separation occurred, as evident from precipitation at 6 h and 16 h. This was attributed to the overreaction of long PNVF chains and the continuous deoxidation of oxygen functional groups on GO nanosheets, as supported by the TEM and zeta potential results (Figure S3 and 4). Interestingly, the quantity of thermal-induced radicals also reached exactly highest at 4 h. Finally, freestanding flexible GF membranes were assembled through vacuum filtration.


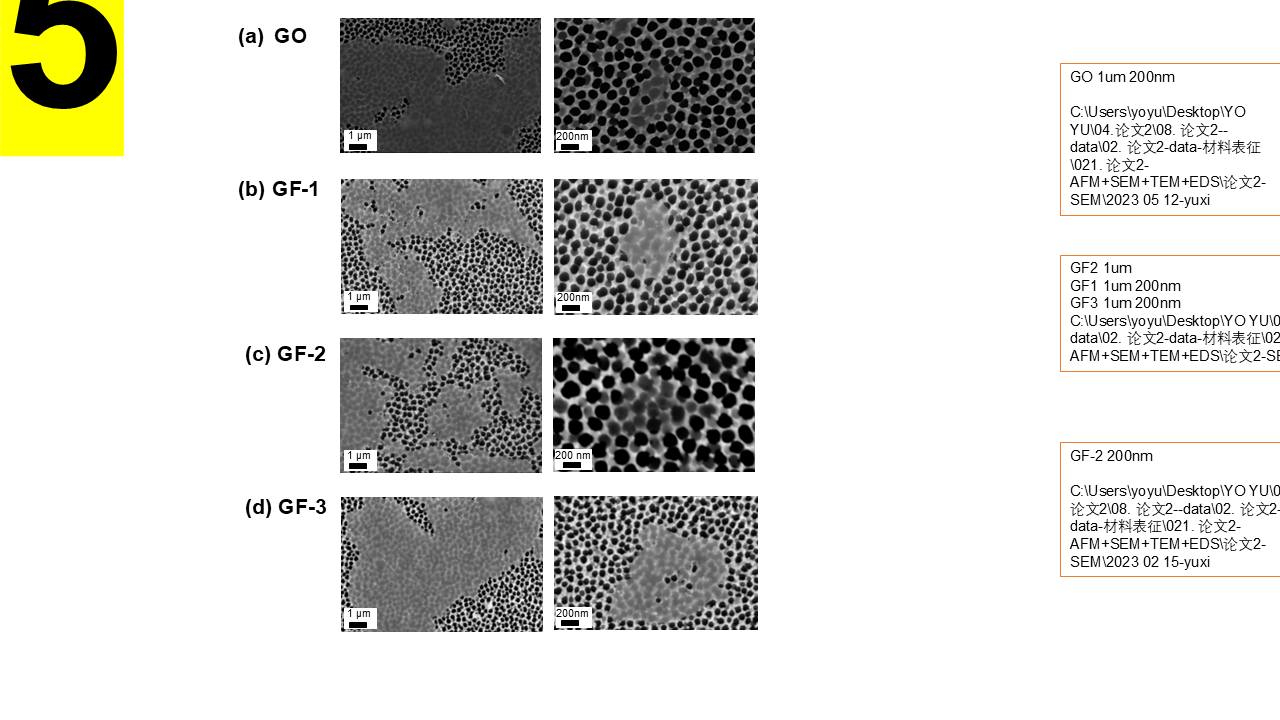


**Figure S5.** SEM images of **(a)** pristine GO nanosheets, **(b)** GF-1, **(c)** GF-2, and **(d)** GF-3 nanosheets.


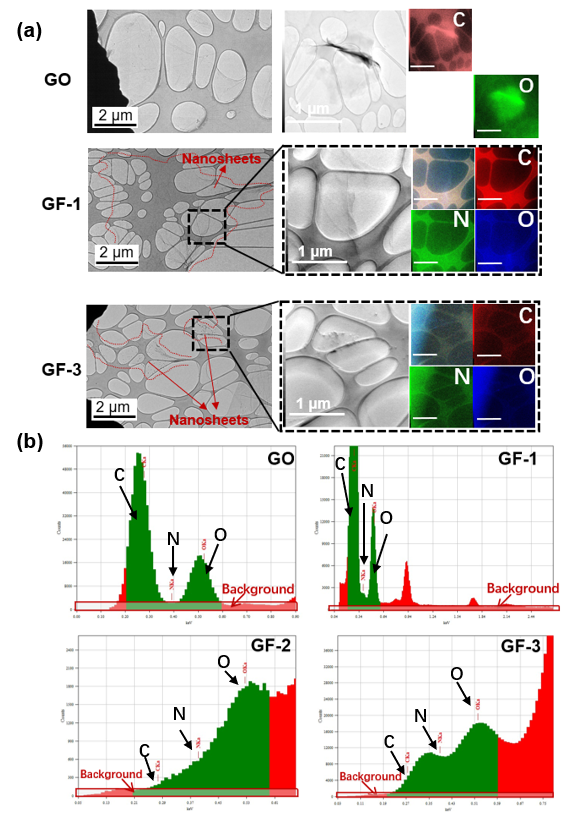


**Figure S6.** **(a)** TEM images and **(b)** EDS mapping of pristine GO nanosheets and as-prepared GF nanosheets.


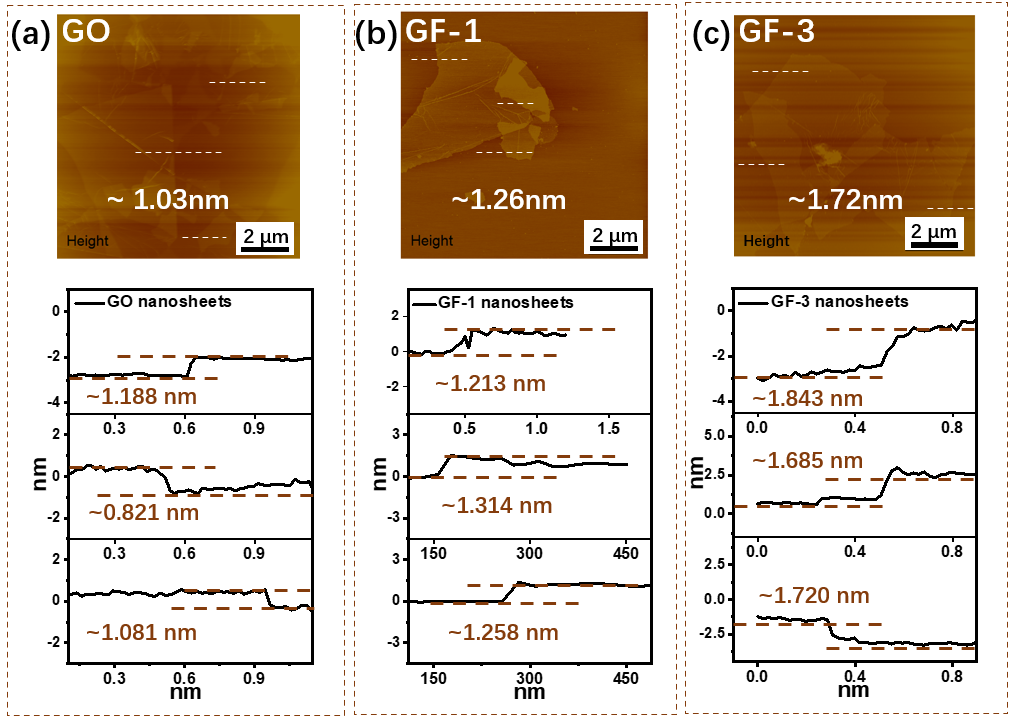


**Figure S7.** AFM images of **(a)** as-prepared GO nanosheets, **(b)** GF-1 nanosheets, and **(c)** GF-3 nanosheets.


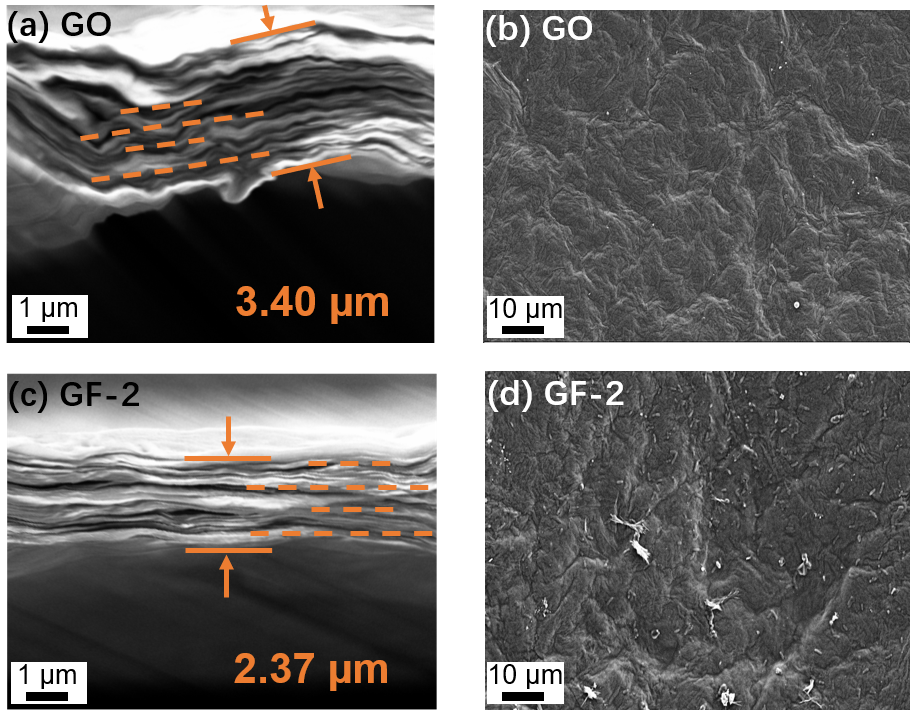


**Figure S8.** SEM images of **(a)** cross section and **(b)** surface of pristine GO membrane, and SEM images of **(c)** cross section and **(d)** surface of GF membranes.


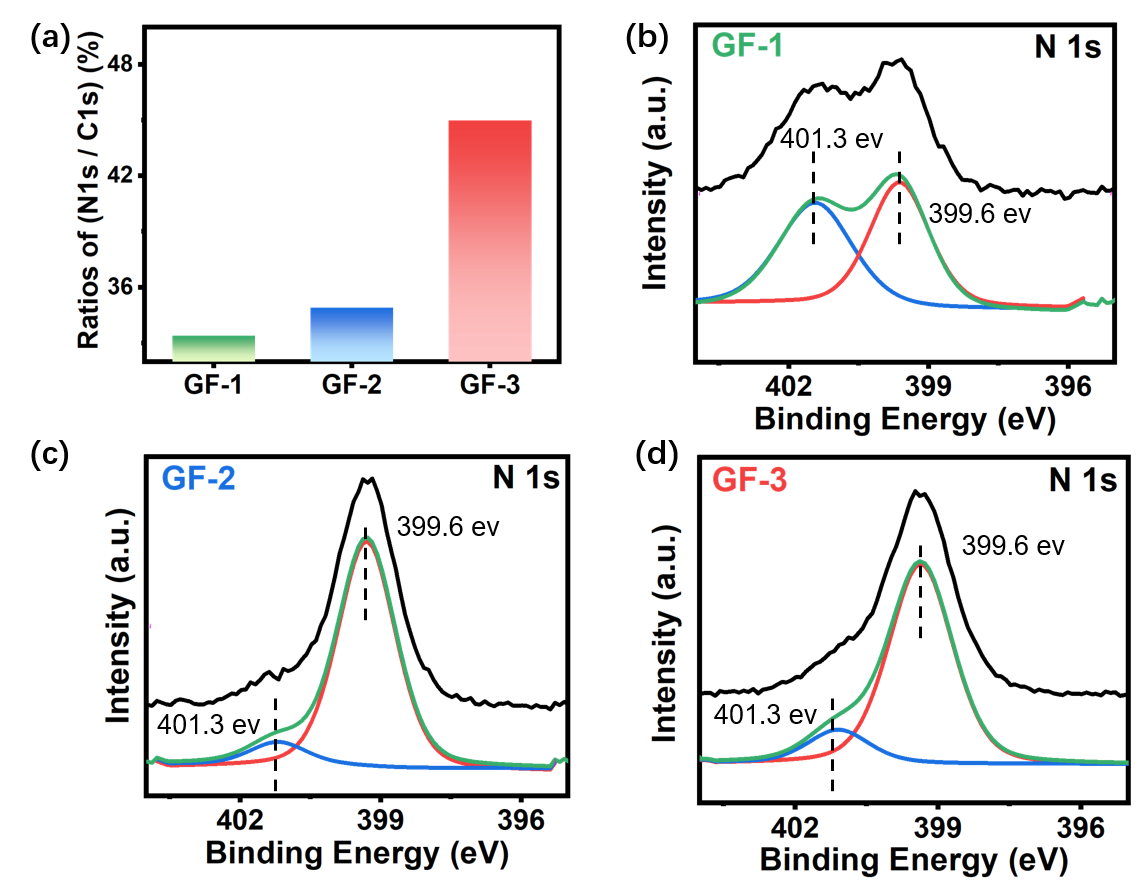


**Figure S9.** **(a)** Relative ratios (N1s/ C1s) of GF membranes, and XPS N1s high solution spectra of as-prepared **(b)** GF-1 membrane, **(c)** GF-2 membrane, and **(d)** GF-3 membrane.


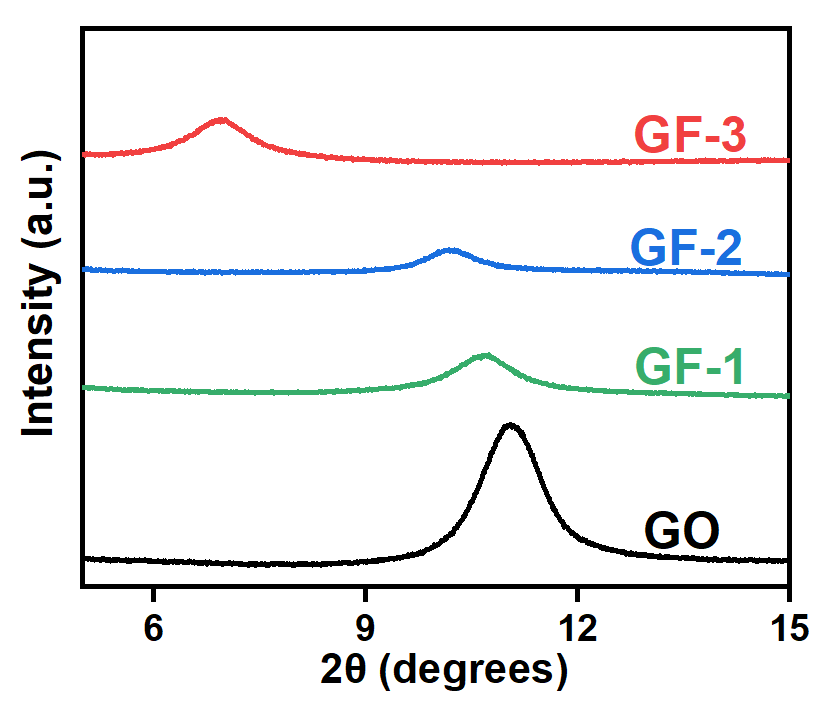


**Figure S10.** XRD spectra of pristine GO and GF membranes.

**Figure S11.** Enlarged region and peak fitting of FTIR spectra for NVF molecules, pristine GO membrane, and GF membranes.


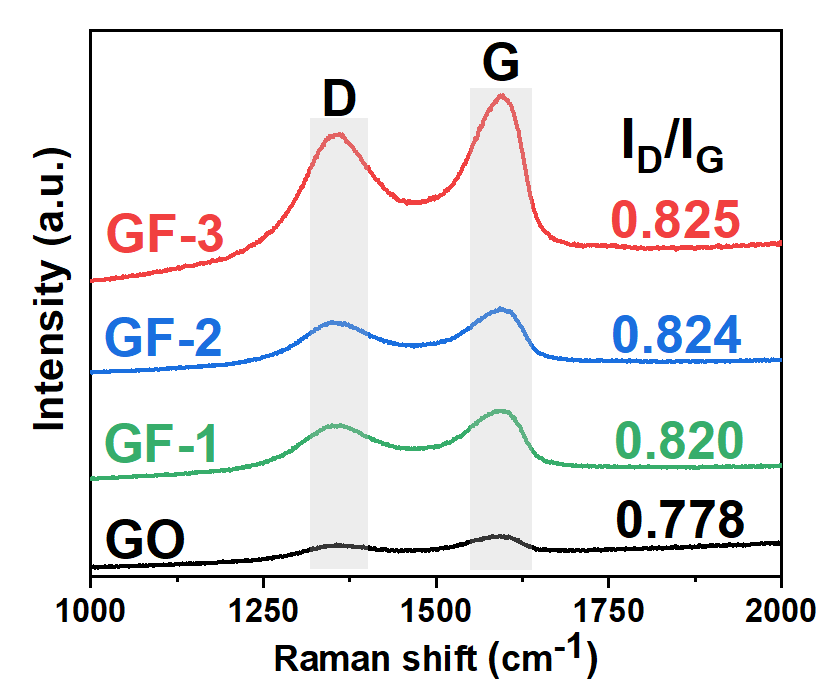


**Figure S12.** Raman spectra of pristine GO and GF membranes.


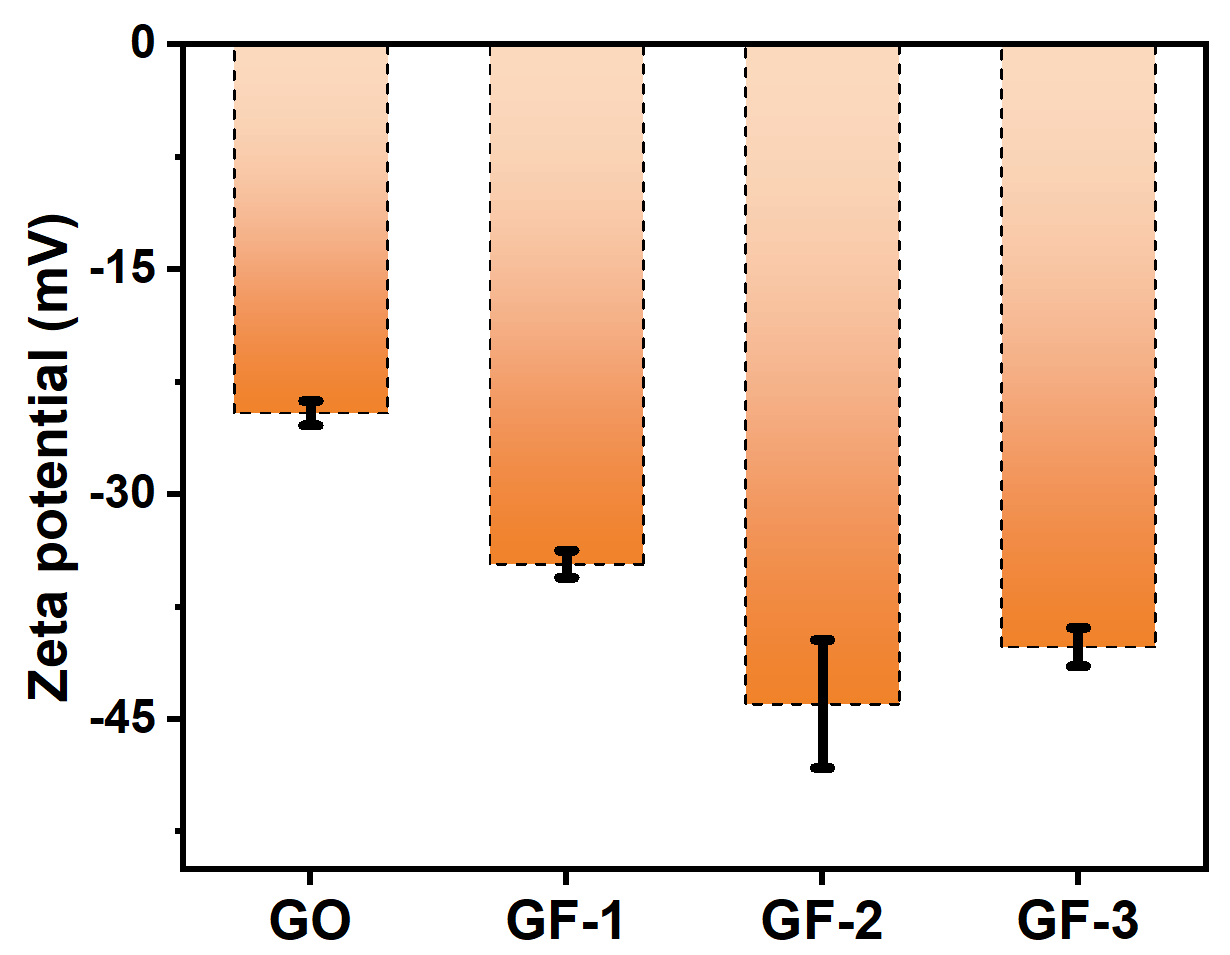


**Figure S13.** Zeta potential values of GO and GF nanosheets.


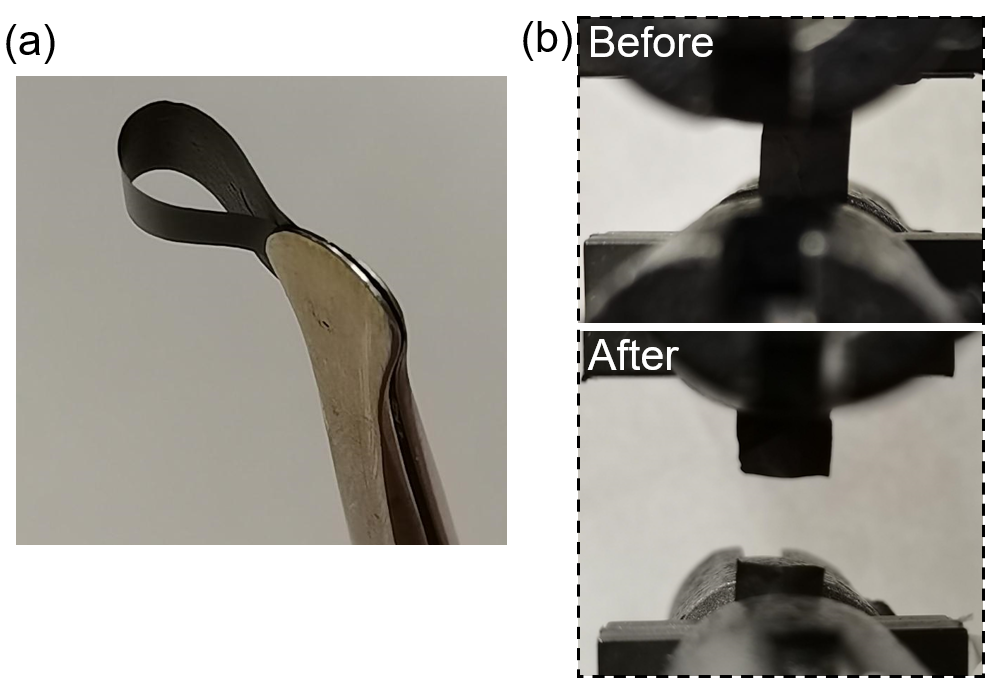


**Figure S14.** Instron tests for the GF membrane. **(a**) the folded GF membrane. **(b-c)** strip before and after fracture from tensile loading.


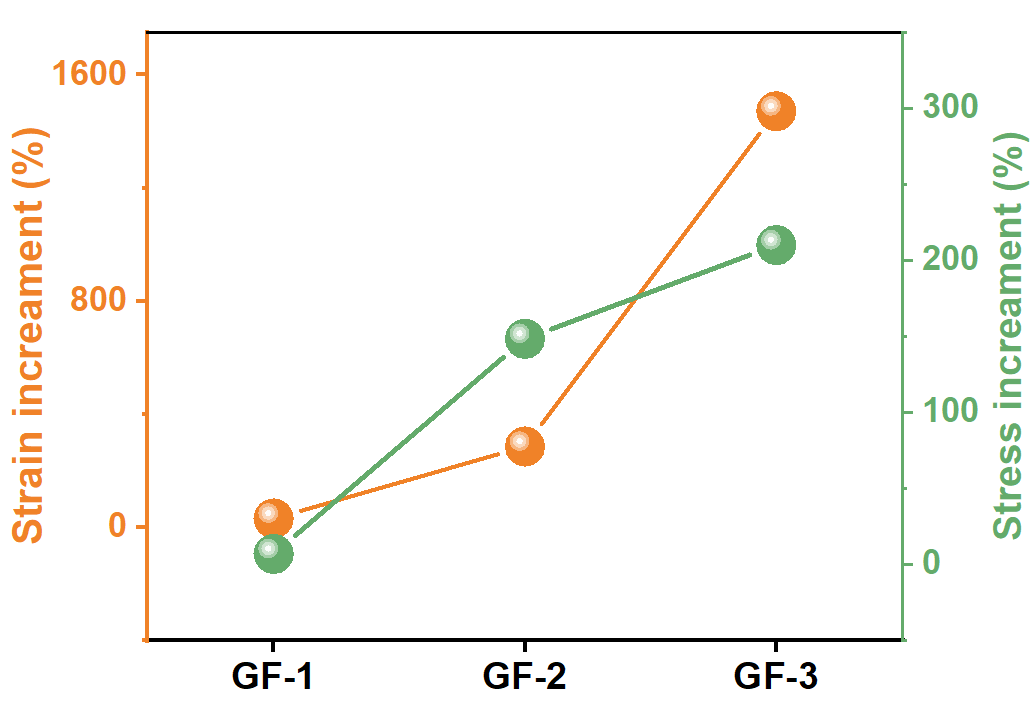


**Figure S15.** Strain and stress increments of GF membranes.


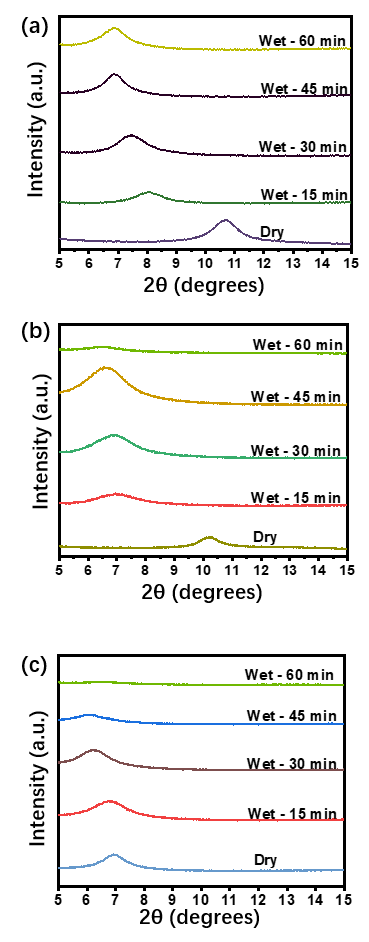


**Figure S16.** XRD of **(a)** GF-1, **(b)** GF-2, and **(c)** GF-3 membranes with different water immersing time.


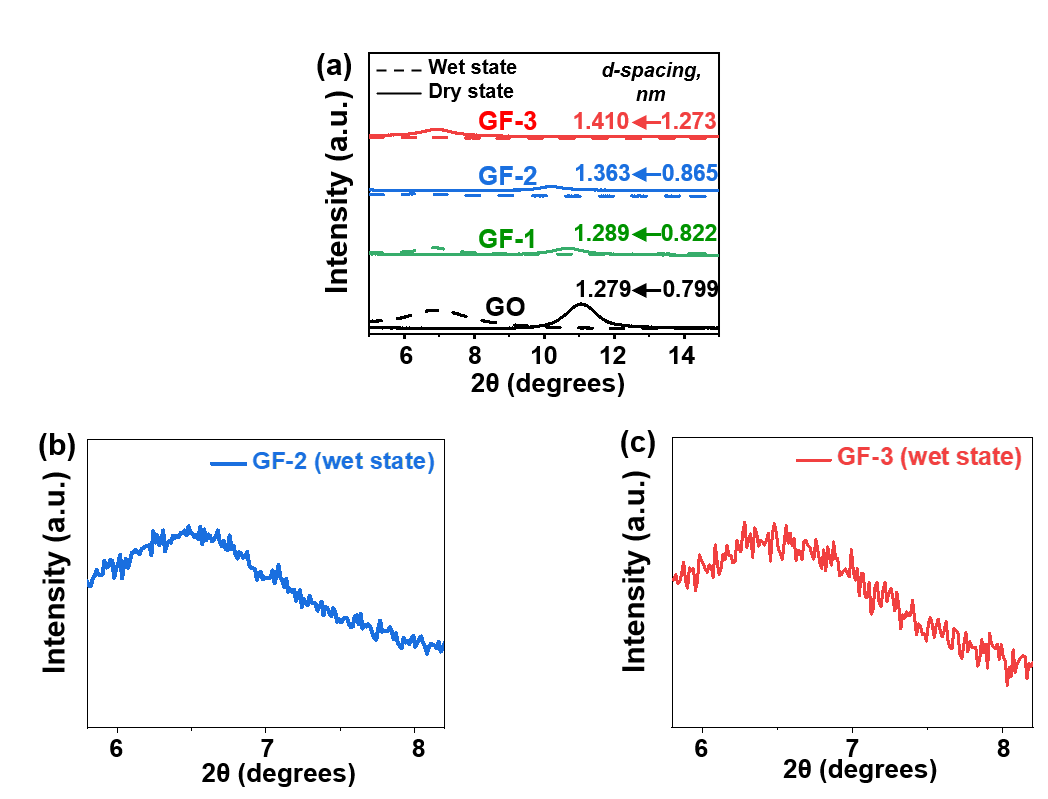


**Figure S17.** XRD patterns of **(a)** GO and GF membranes in the dry and wet state; and enlarged XRD spectra of **(b)** GF-2 and **(c)** GF-3 membranes in the wet stare, with 30 min soaking time.


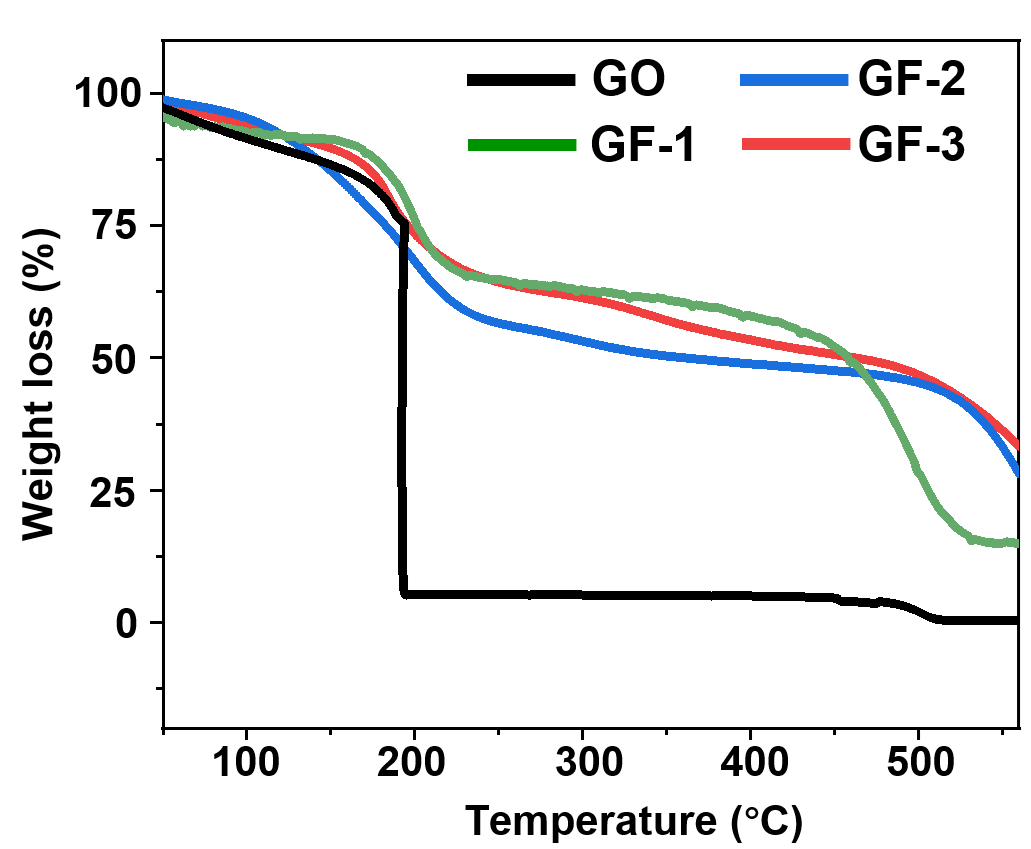


**Figure S18.** TGA spectra of GO and GF membranes.


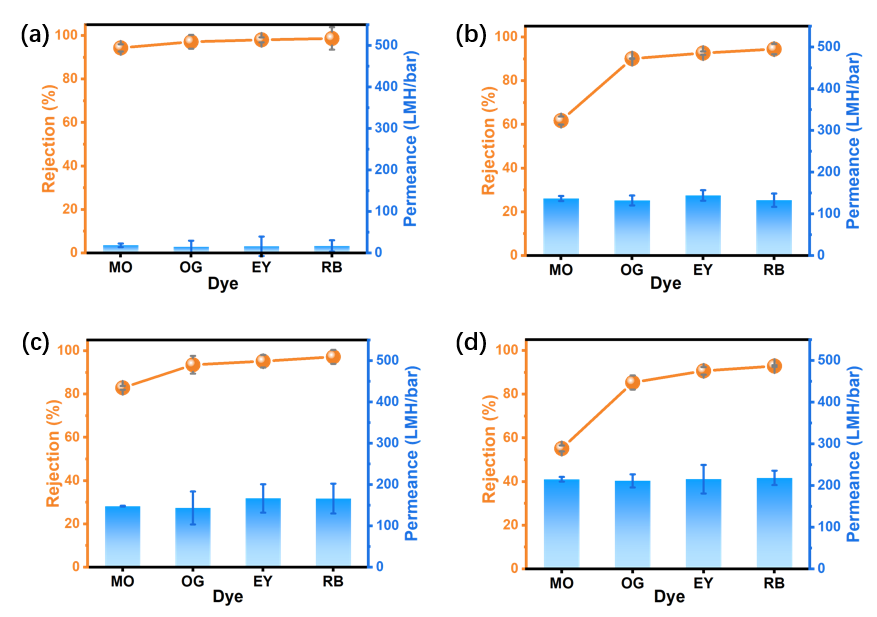


**Figure S19.** Separation performance and water permeance of **(a)** GO membrane, **(b)** GF-1 membrane, **(c)** GF-2 membrane, and **(d)** GF-3 membrane.


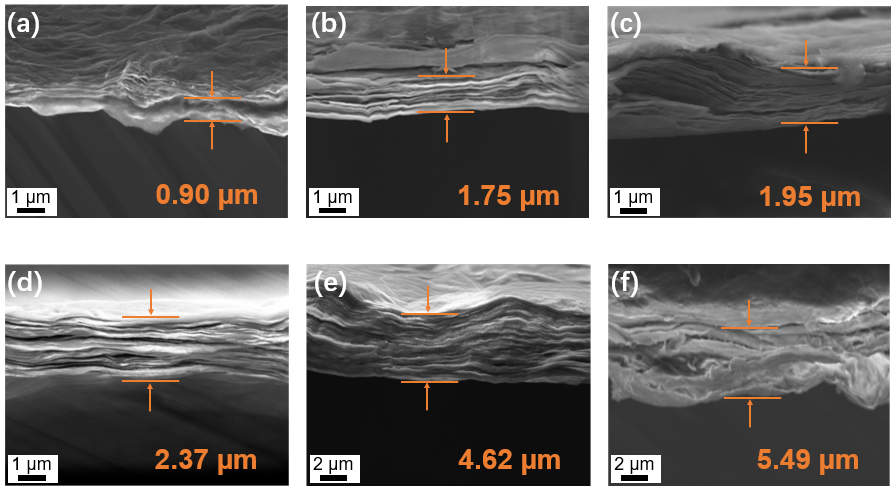


**Figure S20.** SEM for cross section of GF-2 membranes with different loading mass of **(a)** 1.18 mg cm^-2^, **(b)** 1.57 mg cm^-2^, **(c)** 1.97 mg cm^-2^, **(d)** 2.36 mg cm^-2^, **(e)** 3.54 mg cm^-2^, and **(f)** 4.72 mg cm^-2^.

Additional Analysis: GF membranes keep the laminar structure, and the thickness is increased following increasing loading mass. This means PNVF regulates the layer spacing of GO membrane successfully.


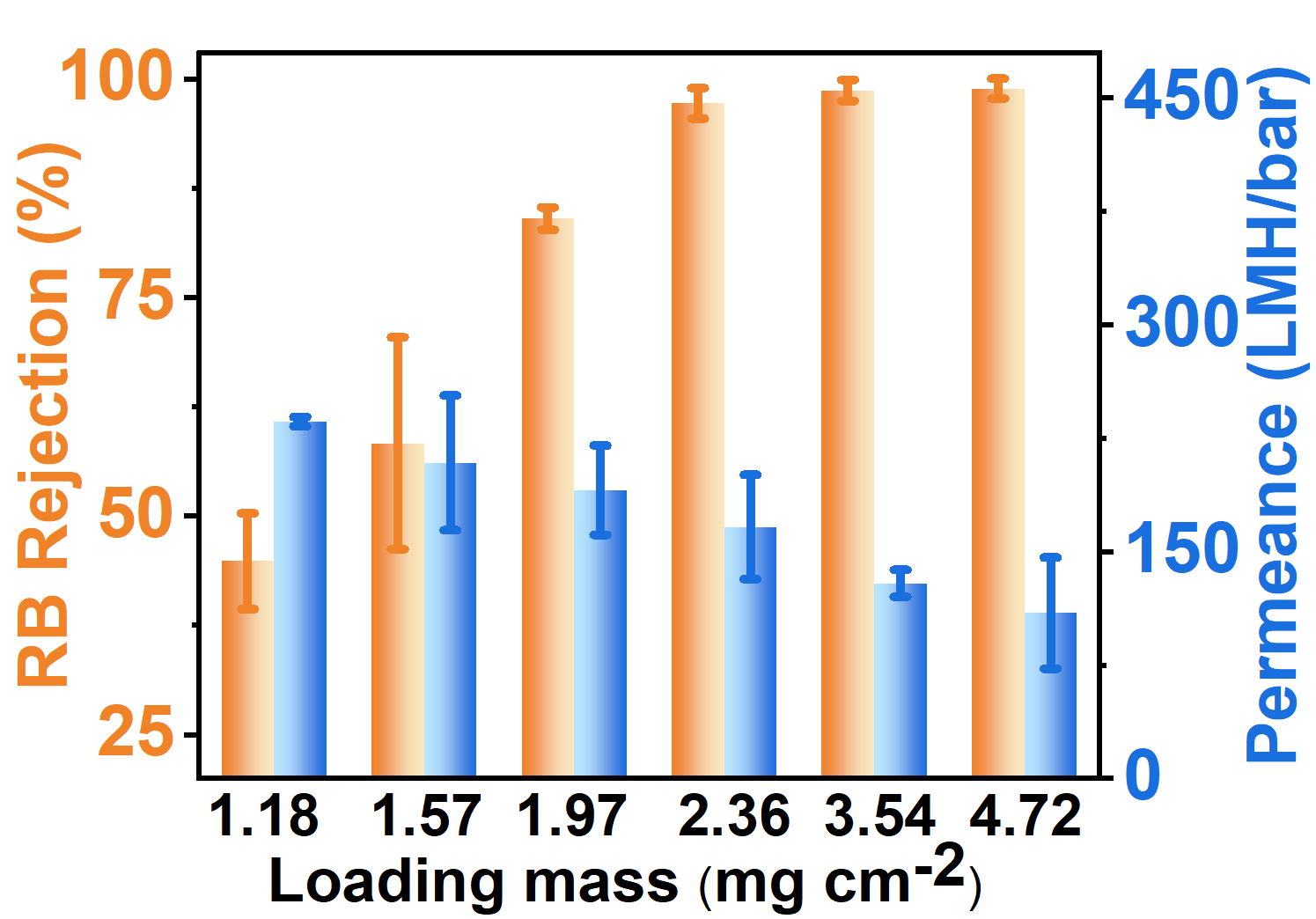


**Figure S21.** Water permeance and RB rejection for GF-2 membranes with different loading mass.


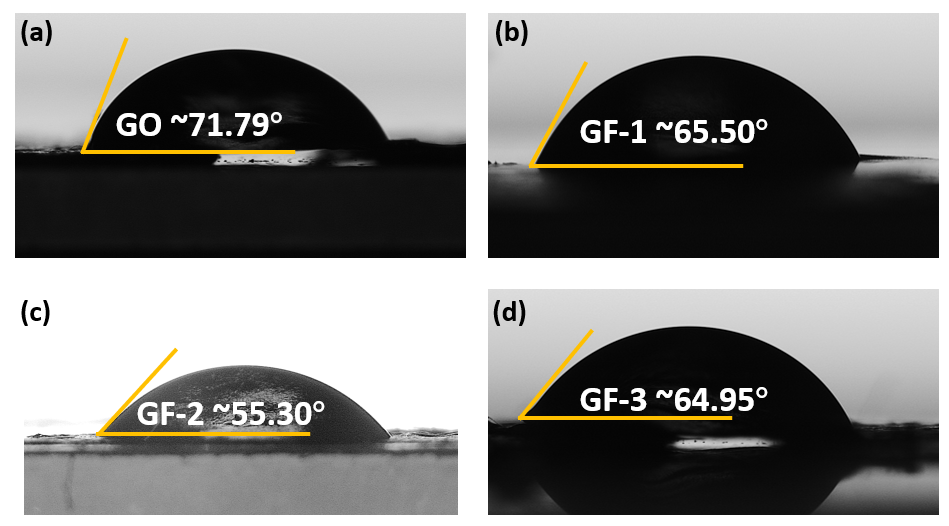


**Figure S22.** Water contact angle images of **(a)** GO, **(b)** GF-1, **(c)** GF-2 and **(d)** GF-3 membranes.


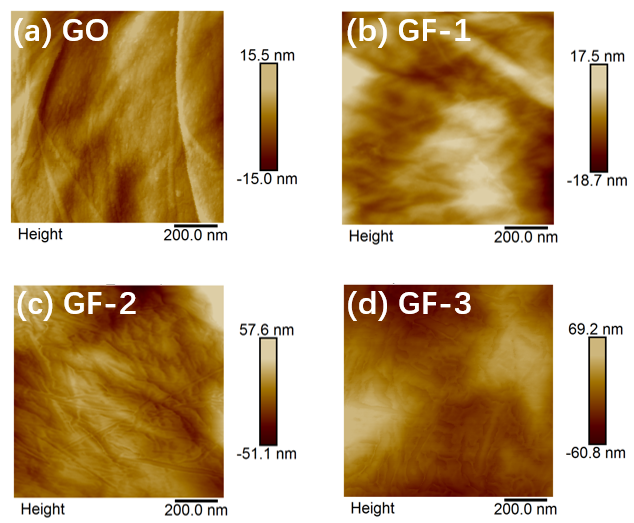


**Figure S23.** AFM images of **(a)** GO, **(b)** GF-1, **(c)** GF-2 and **(d)** GF-3 membranes surface.


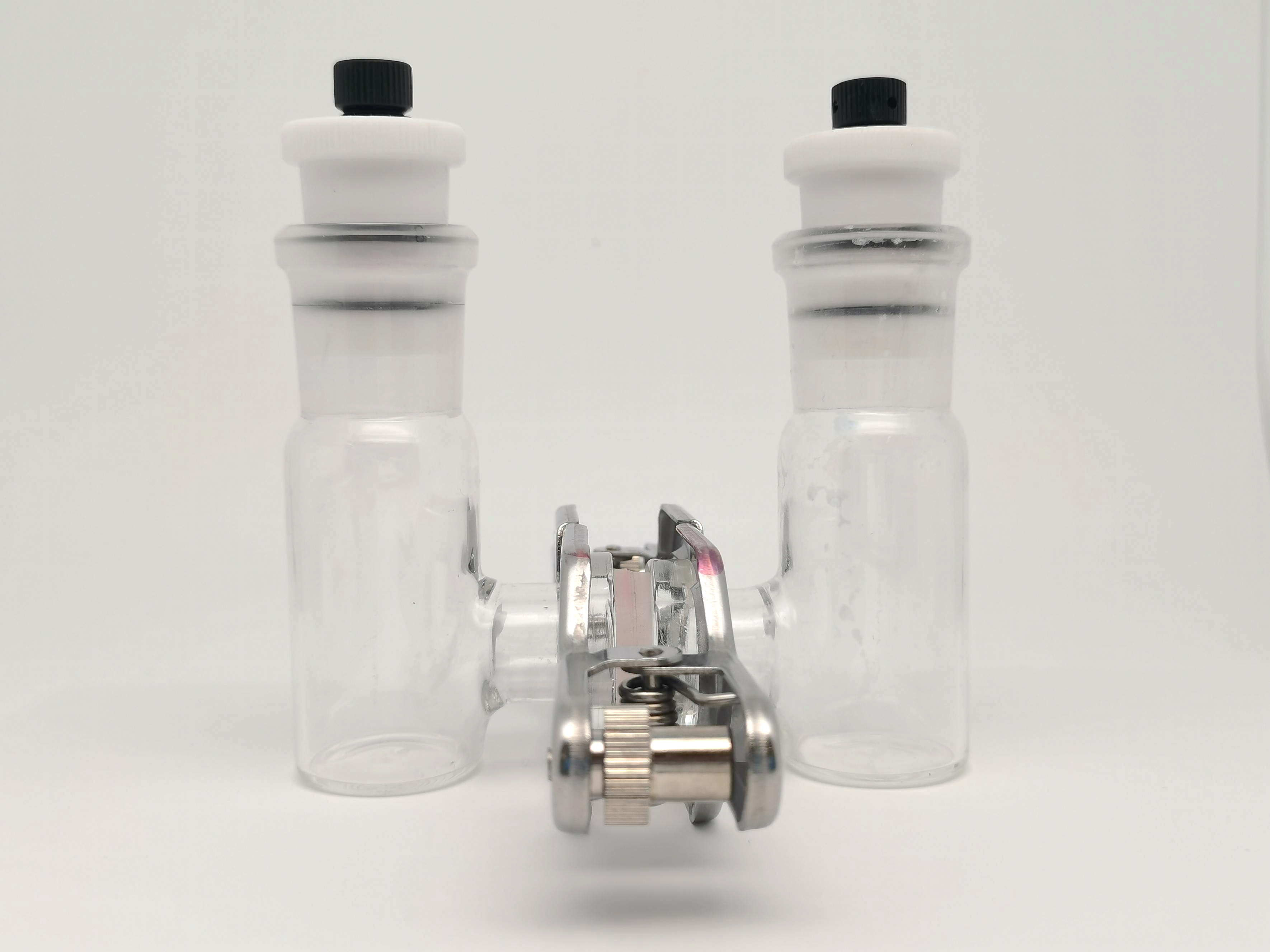


**Figure S24.** Photograph of the home-made H-cell for nanofiltration performance tests.

# **Supplementary Tables**

**Table S1.** Radical contents of GO with different thermal temperatures and times.

| Thermal treatment temperature, °C (4h) | Radical contents of GO | Thermal treatment time, h ( 95 °C) | Radical contents of GO |
| --- | --- | --- | --- |
| 55 °C | 60.30 | **1 h** | 36.92 |
| 95 °C | 93.97 | **2 h** | 81.55 |
| 125 °C | 50.25 | **4 h** | 93.97 |
| 170 °C | 3.672 | **6 h** | 67.24 |
|  |  | **16 h** | 42.25 |

**Table S2.** Comparation of mechanical properties among different membranes and this work.

| Materials | Materials  (full name) | Stress, MPa | Ref. |
| --- | --- | --- | --- |
| BC + GO | bacterial cellulose+graphene oxide | 64.5 | ^[5]^ |
| GO@RO | graphene oxide@polyamide reverse osmosis | 52.37 | ^[6]^ |
| PSF/GO-NH2 | polysulfone/ammonia-functionalized graphene oxide | 5.96 ± 0.52 | ^[7]^ |
| GO/AA/CTA/CA | graphene oxide/acetic acid/cellouse triacetate/cellouse acetate | 39 | ^[8]^ |
| SPEEK/PVA@GO | sulfonated poly(ether ketone) (SPEEK)–polyvinyl alcohol (PVA) matrix@graphene oxide | 68.9 | ^[9]^ |
| PVA/PAA/GO | Polyvinyl alcohol/Polyacrylic acid/graphene oxide | 13.7 ± 1.5 | ^[10]^ |
| polypropylene | polypropylene membranes | 7.32 | ^[11]^ |
| PEI/PVdF | polyetherimide/polyvinylidene fluoride | 48.4 | ^[12]^ |
| PVA-PSSA | poly(vinyl alcohol)-poly(styrene sulfonic acid) | ~4 | ^[13]^ |
| PAN@PVDF-HFP | polyacrylonitrile@poly(vinylidene fluoride-hexafluoro propylene) | ~35 | ^[14]^ |
| Nylon 6 | Nylon 6 | 68.45 | ^[15]^ |
| Nylon 332 | Nylon 332 | 58 | ^[16]^ |
| Nylon-MXD6 | Nylon-MXD6 | 99 | (https://www.mgc.co.jp/eng/products/ac/nmxd6/nature.html) |
| Nylon 66 | Nylon 66 | 77 | (https://www.mgc.co.jp/eng/products/ac/nmxd6/nature.html) |
| GF membrane | GO/NVF membrane | 36~105 | This works |

**Table S3.** Chemical and physical properties of four dye molecules were used in this work.

| **Organic dye** | **Chemical structure** | **Wavelength (nm)** | **Solute**  **Charge** | **Molecular weight** | **C_0_**  **(mg L^-1^)** |
| --- | --- | --- | --- | --- | --- |
| **Rose Bengal**  **(RB)** | 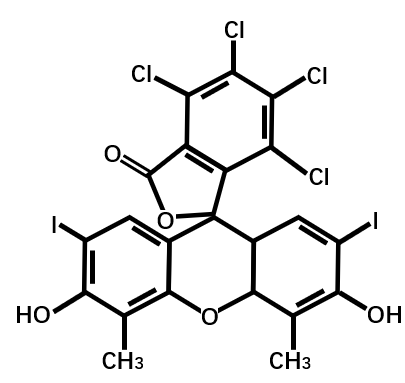 | 575 | Anionic  - | 973.67 Da | 10 |
| **Eosin Yellow**  **(EY)** | 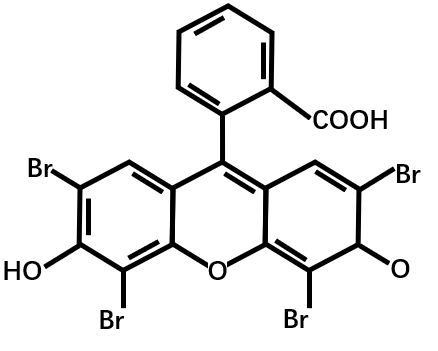 | 510 | Anionic  - | 691.86 Da | 10 |
| **Orange G**  **(OG)** | 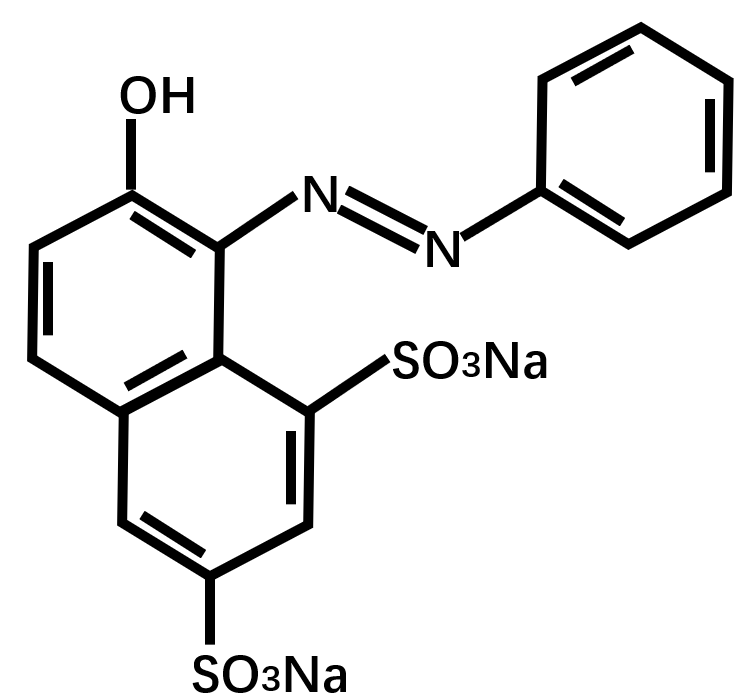 | 196 | Anionic  - | 452.38 Da | 10 |
| **Methyl Orange**  **(MO)** | 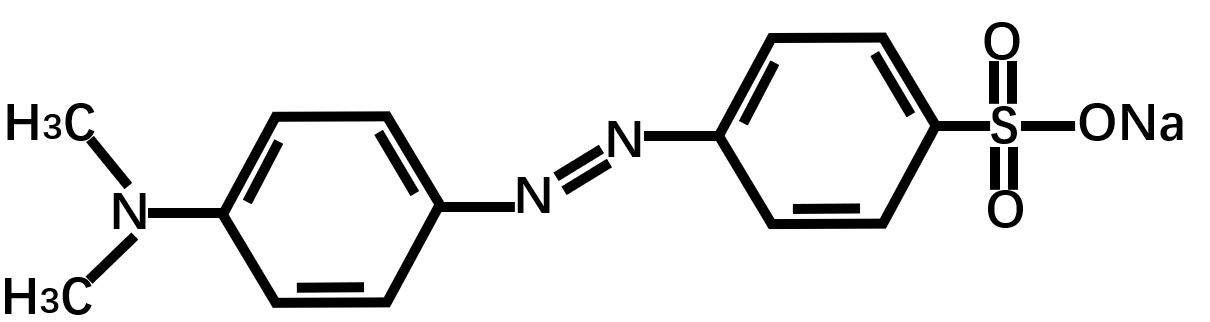 | 465 | Anionic  - | 327.33 Da | 10 |

**Table S4.** Comparison of separation performance among different membranes and this work.

| **Materials** | **Materials**  **(full name)** | **MWCO (Da)** | **Rejection (%)** | **Permeance (LMH bar^-1^)** | **Ref.** |
| --- | --- | --- | --- | --- | --- |
| **GO-TA-Ni** | graphene oxide-Tannic acid-Ni | 319.85 | 92.9 | 71.7 | ^[17]^ |
| **GO/DTiO_2_-PDA-PEI** | graphene oxide/dopamine modified TiO2-polydopamine-polymer ethylene imine | 973.67 | 98 | 41.6 | ^[18]^ |
| **DES/GO** | deep eutectic solvent/graphene oxide | 319.85 | 98.5 | 124.8 | ^[19]^ |
| **Ca/GO-SA_3_** | calcium ions/graphene oxide-sodium alginate | 319.85 | 99 | 38.90 | ^[20]^ |
| **GO/Gr** | graphene oxide/graphene | 696.665 | ≥97.3 | 20 | ^[21]^ |
| **GO@TiO_2_** | graphene oxide@TiO_2_ | 461.38 | >98 | 3.6 | ^[22]^ |
| **GO/TiO_2_** | graphene oxide/TiO_2_ | 288.78 | 97 | 89.6 | ^[23]^ |
| **GO-PPD** | graphene oxide-P-Phenylenediamine | 319.85 | 99 | 42 | ^[24]^ |
| **GQDs** | graphene quantum dots | 960.81 | >95 | 128.1 | ^[25]^ |
| **NbN-GO** | niobate nanosheet-graphene oxide | 960.81 | >99 | 20 | ^[26]^ |
| **PEI-mica/CNFs** | polyethylenimine-mica nanosheets/cellulose nanofibers | 696.665 | >99 | 62.18 | ^[27]^ |
| **GO-PDA/PES** | graphene oxide-polydopamine/polyether sulfone | 452.38 | 95 | 85 | ^[28]^ |
| **MXene/CNT** | MXene/carbon nanotubes | 452.38 | >96 | 30.4 | ^[29]^ |
| **GO@PDA/PES** | graphene oxide@polydopamine/polyethersulfone | 696.665 | >99 | 49.5 | ^[30]^ |
| **G-CNTm** | graphene-carbon nanotubes | 319.85 | >96 | 11.3 | ^[31]^ |
| **This work** | **Solute** | **MWCO (Da)** | **Rejection (%)** | **Permeance (LMH bar^-1^)** |  |
| GF-1 | MO | 639.36 | >61.79±3.18 | 136.73±14.45 |  |
| GF-1 | OG |  | >90.16±0.11 | 132.09±11.92 |  |
| GF-1 | EY |  | >92.71±4.12 | 144.02±39.87 |  |
| GF-1 | RB |  | >94.51±3.25 | 132.70±15.70 |  |
| GF-2 | MO | 432.74 | >82.94±1.15 | 147.79±23.55 |  |
| GF-2 | OG |  | >93.58±0.82 | 143.35±12.55 |  |
| GF-2 | EY |  | >95.22±2.92 | 166.53±34.45 |  |
| GF-2 | RB |  | >97.25±1.72 | 166.18±34.26 |  |
| GF-3 | MO | 730.01 | >55.16±5.17 | 215.09±13.67 |  |
| GF-3 | OG |  | >85.41±2.37 | 211.32±16.02 |  |
| GF-3 | EY |  | >90.71±3.22 | 215.33±36.12 |  |
| GF-3 | RB |  | >92.93±0.41 | 218.48±17.23 |  |

**Table S5.** The surface roughness of GO and GF membrane.

|  | **GO** | **GF-1** | **GF-2** | **GF-3** |
| --- | --- | --- | --- | --- |
| **Rq (nm)** | 4.62 | 5.30 | 13.4 | 20.3 |
| **Ra (nm)** | 3.65 | 4.30 | 10.2 | 16.7 |

# **References**

[1] W. S. Hummers, R. E. Offeman, *J. Am. Chem. Soc.* **1958**, *80*, 1339–1339.

[2] Y. Qian, J. Shang, D. Liu, G. Yang, X. Wang, C. Chen, L. Kou, W. Lei, *J. Am. Chem. Soc.* **2021**, *143*, 5080–5090.

[3] S. Marbach, L. Bocquet, *Chem. Soc. Rev.* **2019**, *48*, 3102–3144.

[4] Z. Komeily-Nia, J.-Y. Chen, B. Nasri-Nasrabadi, W.-W. Lei, B. Yuan, J. Zhang, L.-T. Qu, A. Gupta, J.-L. Li, *Phys. Chem. Chem. Phys.* **2020**, *22*, 3112–3121.

[5] Q. Fang, X. Zhou, W. Deng, Z. Zheng, Z. Liu, *Sci. Rep.* **2016**, *6*, 33185.

[6] M. Y. Ashfaq, M. A. Al-Ghouti, N. Zouari, *Carbon* **2020**, *166*, 374–387.

[7] M. Ionita, A. M. Pandele, L. E. Crica, A. C. Obreja, *High Perform. Polym.* **2016**, *28*, 181–188.

[8] D. F. Ahmed, H. Isawi, N. A. Badway, A. A. Elbayaa, H. Shawky, *Arab. J. Chem.* **2021**, *14*, 102995.

[9] J. L. Reyes-Rodriguez, J. Escorihuela, A. García-Bernabé, E. Giménez, O. Solorza-Feria, V. Compañ, *RSC Adv.* **2017**, *7*, 53481–53491.

[10] Q. Chen, X. Yan, K. Chen, C. Feng, D. Wang, X. Li, X. Zhao, Z. Chai, Q. Wang, D. Zhang, H. Zeng, *J. Mater. Chem. B* **2023**, *11*, 1713–1724.

[11] G. M. Wu, S. J. Lin, J. H. You, C. C. Yang, *Mater. Chem. Phys.* **2008**, *112*, 798–804.

[12] J. Chae, S. Park, D. Y. Kim, H.-I. Joh, J. M. Kim, S. Lee, S. M. Jo, *Polymers* **2018**, *10*, 436.

[13] A. K. Sahu, G. Selvarani, S. Pitchumani, P. Sridhar, A. K. Shukla, N. Narayanan, A. Banerjee, N. Chandrakumar, *J. Electrochem. Soc.* **2008**, *155*, B686.

[14] S. Yang, W. Ma, A. Wang, J. Gu, Y. Yin, *RSC Adv.* **2018**, *8*, 23390–23396.

[15] S. Krishna, C. M. Patel, *Mech. Mater.* **2020**, *143*, 103318.

[16] A. Yankin, Y. Alipov, A. Temirgali, G. Serik, S. Danenova, D. Talamona, A. Perveen, *Polymers* **2023**, *15*, 3043.

[17] X. Kang, Y. Cheng, Y. Wen, J. Qi, X. Li, *J. Hazard. Mater.* **2020**, *400*, 123121.

[18] Y. Xu, G. Peng, J. Liao, J. Shen, C. Gao, *J. Membr. Sci.* **2020**, *601*, 117727.

[19] N. Mehrabi, H. Lin, N. Aich, *Chem. Eng. J.* **2021**, *412*, 128577.

[20] J. Yu, Y. Wang, Y. He, Y. Gao, R. Hou, J. Ma, L. Zhang, X. Guo, L. Chen, *Sep. Purif. Technol.* **2021**, *276*, 119348.

[21] P. Zhang, Y. Wang, P. Li, X. Luo, J. Feng, H. Kong, T. Li, W. Wang, X. Duan, Y. Liu, M. Li, *Sci. Total Environ.* **2022**, *827*, 154175.

[22] L. Zhu, M. Wu, B. Van der Bruggen, L. Lei, L. Zhu, *Sep. Purif. Technol.* **2020**, *242*, 116770.

[23] R. Han, P. Wu, *J. Mater. Chem. A* **2019**, *7*, 6475–6481.

[24] K. Zhou, C. Guo, F. Gan, J. H. Xin, H. Yu, *J. Colloid Interface Sci.* **2023**, *640*, 261–269.

[25] Y. Li, R. Bi, Y. Su, Y. Li, C. Yang, X. You, J. Shen, J. Yuan, R. Zhang, Z. Jiang, *Sep. Purif. Technol.* **2021**, *263*, 118372.

[26] M. Kunimatsu, K. Nakagawa, T. Yoshioka, T. Shintani, T. Yasui, E. Kamio, S. C. E. Tsang, J. Li, H. Matsuyama, *J. Membr. Sci.* **2020**, *595*, 117598.

[27] Q. Shi, N. Zhang, D. Wang, J. Zhang, Y. Li, Z. Wang, *Desalination* **2023**, *551*, 116410.

[28] C. Wang, Z. Li, J. Chen, Y. Yin, H. Wu, *Appl. Surf. Sci.* **2018**, *427*, 1092–1098.

[29] G. Yi, L. Du, G. Wei, H. Zhang, H. Yu, X. Quan, S. Chen, *J. Membr. Sci.* **2022**, *658*, 120719.

[30] C. Wang, Y. Feng, J. Chen, X. Bai, L. Ren, C. Wang, K. Huang, H. Wu, *J. Taiwan Inst. Chem. Eng.* **2020**, *110*, 153–162.

[31] Y. Han, Y. Jiang, C. Gao, *ACS Appl. Mater. Interfaces* **2015**, *7*, 8147–8155.
